# Association between oral microbial dysbiosis and poor functional outcomes in stroke-associated pneumonia patients

**DOI:** 10.1186/s12866-023-03057-8

**Published:** 2023-10-24

**Authors:** Yueran Ren, Jingru Liang, Xiao Li, Yiting Deng, Sanping Cheng, Qiheng Wu, Wei Song, Yan He, Jiajia Zhu, Xiaomei Zhang, Hongwei Zhou, Jia Yin

**Affiliations:** 1 Department of Neurology, Nanfang Hospital, Southern Medical University, Guangzhou, Guangdong China; 2 Microbiome Medicine Center, Department of Laboratory Medicine, Zhujiang Hospital, Southern Medical University, Guangzhou, Guangdong China

**Keywords:** Oral microbiota, Dysbiosis, Stroke-associated Pneumonia, Prognosis, Biomarker

## Abstract

**Background:**

Despite advances in our understanding of the critical role of the microbiota in stroke patients, the oral microbiome has rarely been reported to be associated with stroke-associated pneumonia (SAP). We sought to profile the oral microbial composition of SAP patients and to determine whether microbiome temporal instability and special taxa are associated with pneumonia progression and functional outcomes.

**Methods:**

This is a prospective, observational, single-center cohort study that examined patients with acute ischemic stroke (AIS) who were admitted within 24 h of experiencing a stroke event. The patients were divided into three groups based on the occurrence of pneumonia and the use of mechanical ventilation: nonpneumonia group, SAP group, and ventilator-associated pneumonia (VAP) group. We collected oral swabs at different time points post-admission and analyzed the microbiota using 16 S rRNA high-throughput sequencing. The microbiota was then compared among the three groups.

**Results:**

In total, 104 nonpneumonia, 50 SAP and 10 VAP patients were included in the analysis. We found that SAP and VAP patients exhibited significant dynamic differences in the diversity and composition of the oral microbiota and that the magnitude of this dysbiosis and instability increased during hospitalization. Then, by controlling the potential effect of all latent confounding variables, we assessed the changes associated with pneumonia after stroke and explored patients with a lower abundance of *Streptococcus* were more likely to suffer from SAP. The logistic regression analysis revealed that an increase in specific taxa in the phylum *Actinobacteriota* was linked to a higher risk of poor outcomes. A model for SAP patients based on oral microbiota could accurately predict 30-day clinical outcomes after stroke onset.

**Conclusions:**

We concluded that specific oral microbiota signatures could be used to predict illness development and clinical outcomes in SAP patients. We proposed the potential of the oral microbiota as a non-invasive diagnostic biomarker in the clinical management of SAP patients.

**Clinical Trial registration:**

NCT04688138. Registered 29/12/2020, https://clinicaltrials.gov/ct2/show/NCT04688138.

**Supplementary Information:**

The online version contains supplementary material available at 10.1186/s12866-023-03057-8.

## Introduction

Stroke-associated pneumonia (SAP) is one of the most frequent complications of stroke [1–3]. Additionally, ventilator-associated pneumonia (VAP) is a common and serious clinical issue among critically ill stroke patients. Notably, AIS patients who have SAP or VAP tend to have poorer outcomes, including longer hospital stays, higher costs, increased discharge dependency, and a greater risk of death [2, 4, 5]. Despite this, there has been limited progress in the prevention of SAP. Therefore, we must urgently enhance our understanding of the unidentified factors that are associated with or even cause SAP.

Substantial growth in the body of research on the human gut microbiota and its potential role in promoting stroke has occurred during the past decade [6–8]. Gut microbiota dysbiosis has been reported to be associated with SAP [9, 10]. However, in recent years, there has been an increasing report on the disruption of the oral microbiome specifically, which has been found to promote stroke and complications following stroke [11–13]. The oral hygiene and cleanliness of stroke patients’ mouth and teeth may contribute to the development of SAP as highly diverse microbial communities [14], including anaerobes and indigenous oral bacteria, can result in pneumonia. In previous studies, SAP patients were found to exhibit oral microbiota dysbiosis [15–17], and the domination of certain oral pathogens has been identified as a risk factor for adverse outcomes in VAP [18–20]. However, there is limited information available regarding the changes that occur in the oral microbiota during stroke and whether the characteristics of the oral microbiome are linked to poor clinical outcomes among patients in stroke units.

Notably, some clinical trials have explored the impacts of heightened oral cleanliness care on decreasing SAP and VAP rate [14, 21–24]. However, there are still challenges and uncertainties surrounding the assessment and care of oral hygiene in stroke units. Researchers and clinicians do not agree on the best instrument or optimal timing for conducting oral assessments [21].

Therefore, oral microbial studies concerning SAP should aim to investigate potential oral microbial biomarkers and establish larger observational patient cohorts. In this prospective observational study, we investigated alterations in the composition of oral microbiota at various time points after admission and their association with SAP. The objective was to assess the progression and recovery of the microbial community shift after a stroke. Additionally, we aimed to determine the impact of oral microbial dysbiosis on patient outcomes and explore predictive variables using various features of the oral microbiota assessment.

## Methods

### Ethics statement and participants

This study was approved by the Ethics Committee of Nanfang Hospital, Southern Medical University (NFEC-2020-169) and registered at http://clinicaltrials.gov (NCT04688138). Informed consent was obtained from all patients or their legal representatives. This research was conducted in accordance with the Declaration of Helsinki. Acute stroke patients were recruited from the Department of Neurology of Nanfang Hospital of Southern Medical University (Guangzhou, China) from December 2020 to September 2021. The inclusion criteria were as follows: (1) age > 18 years and (2) admission within 24 h of ischemic stroke onset. Exclusion criteria were as follows: (1) antibiotic, prebiotic, or probiotic use within three months before admission; (2) admission after 24 h of stroke onset; (3) active infection within the 2 weeks before admission; (4) history of oral and respiratory cancer or advanced cancer; (5) failure to offer oral swab samples during hospitalization; and (6) the use of an immunosuppressant or a history of systemic diseases like cirrhosis, renal failure, and hematologic conditions.

### Clinical data and sample collection

Clinical data and treatment records were collected by professional trained neurologists. As previously reported, the stroke risk factor assessment was carried out [10]. The Glasgow Coma Scale (GCS), Acute Physiology and Chronic Health Evaluation (APACHE-II), Sequential Organ Failure Assessment (SOFA) scores, modified Rankin Scale (mRS) and National Institutes of Health Stroke Scale (NIHSS) were recorded by specialized neurocritical care physicians at admission. Fasting blood samples were analyzed in the Clinical Laboratory of Nanfang Hospital, and routine blood parameters were estimated. Upon enrollment, we noninvasively collected oral biospecimens from the the buccal mucosa and gingiva using a sterile cotton swab, placed them in an Eppendorf tube with 1 mL phosphate-buffered saline and rapidly transported and stored them at -80 ℃ [25]. Specimen collection was performed at different time points after enrollment. However, the number of specimens at each time point has varied due to premature discharge, early transfer of patients, or early death. The quantity of specimens at each time point has been depicted in Fig. 2A.

### Definitions of SAP and VAP and functional outcomes

SAP was recorded as the improvement of lower respiratory infections during the initial 7 days after stroke onset [1, 2]. Furthermore, existing diagnostic criteria for VAP include that patients receive mechanical ventilation in the first 7 days after stroke [4, 26–30]. Additional diagnosis criteria in the Supplemental Material 1.

The clinical cohort’s stroke outcomes, including hospitalization duration, discharge NIHSS score, 30-day mortality, and mRS scores at 30 and 90 days, were examined through phone follow-up with patients or their caregivers or through medical records obtained during hospitalization or from outpatient clinics.

### 16 S rRNA sequencing and analysis

DNA was extracted using the Mabio® Bacterial DNA Extraction Mini Kit for oral swab samples. The concentration and purity were measured using a NanoDrop One (Thermo Fisher Scientific, MA, USA). Amplify different regions of 16 S rRNA (V3-V4) using specific primers (338 F and 806R) with a 12 bp barcode. Using the NEBNext® Ultra™ II DNA Library Prep Kit for Illumina® (New England Biolabs, MA, USA) to generate the sequencing libraries, and added index codes. The Qubit@ 2.0 Fluorometer (Thermo Fisher Scientific, MA, USA) was used to assess the quality of the library. Finally, the library was sequenced on the Illumina Nova6000 platform and a paired end reading of 250 bp was generated (Guangdong Magigene Biotechnology Co., Ltd. Guangzhou, China). Chimeric sequences were removed and sequencing noise was detected using the DADA2 method. With 99% similarity, the sequences were grouped into amplicon sequence variants (ASVs). Using the vsearch alignment method and the SILVA v132 database, the q2-feature-classifier plugin was used to classify the sequences according to their taxonomic information. Please refer to the supplementary material 1 for more information on the content of the 16 S rRNA sequencing analysis.

### Statistical analyses

Data were analyzed using IBM’s SPSS 25.0 statistical software package. The study population was described by demographic and clinical variables. We used the mean (standard deviation) to express measurement data that conformed to a normal distribution, the median (interquartile range) to express measurement data that had a skewed distribution, and a percentage to express numerical data. The Kruskal–Wallis test and Mann–Whitney U test were also performed to determine statistical significance. Spearman correlation analysis was used for correlation tests. Univariate and multivariate logistic regression analyses were performed, and odds ratios (ORs) and 95% CIs were calculated to identify the risk factors for clinical outcomes. Furthermore, univariate and multivariate Cox proportional hazards models were used to evaluate the association between the abundance of specific taxa and SAP. Predictive performance for clinical outcomes was assessed by comparing receiver operator characteristic (ROC) curves.

## Results

### Demographic and clinical data

**Fig. 1:**
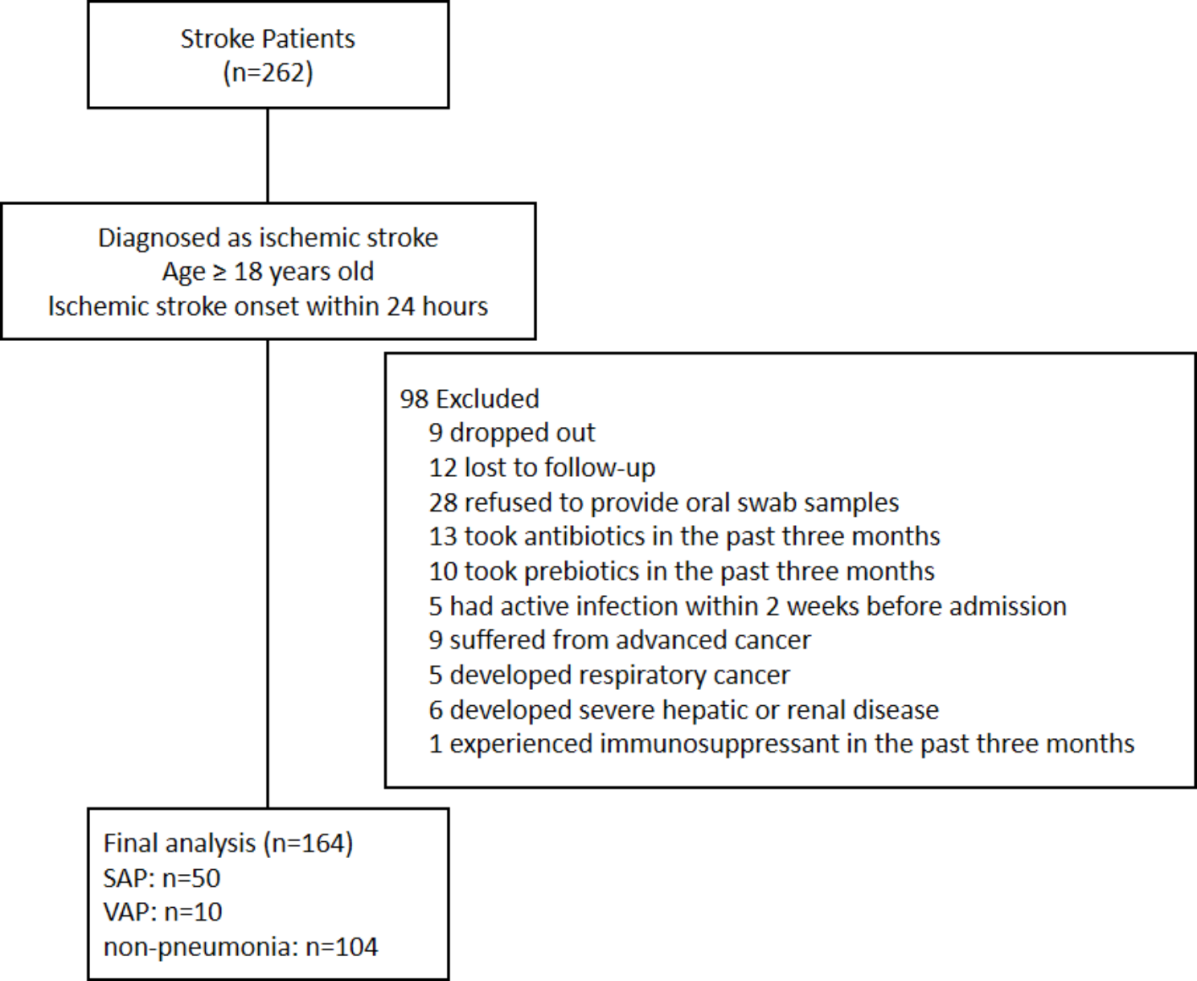
Flow diagram of the patient selection process

Between December 30, 2020, and September 15, 2021, we recruited 164 AIS patients admitted within 24 h after stroke events and divided them into three groups according to the presence of pneumonia and the use of ventilators. Figure 1 depicts the patient selection process’s flow diagram. Demographic and clinical data for the participants are introduced in Table 1. We found no significant difference among the three groups in terms of sex, history of stroke, or smoking or drinking habits. Patients in the SAP group were older than patients without pneumonia. Stroke severity was significantly higher in the SAP and VAP groups. Furthermore, the SAP and VAP groups suffered significantly more often from dysphagia and enteral nutrition than the nonpneumonia group. Regarding revascularization treatments for AIS and oral hygiene care, there was no significant difference among the three groups. The SAP and VAP groups showed a longer duration of ICU hospitalization, higher rates of 30-day mortality and more severe 90-day mRS score, indicating that these AIS patients who developed pneumonia during the hospital stay suffered from a poor stroke prognosis. For dental date, there were no significant differences in the percentage of caries, missing teeth, residual root, periodontitis or calculus between the three groups (Table 2).

**Table 1:** Baseline characteristics of patients among the three groups

	nonpneumonia(n = 104)	SAP (n = 50)	VAP (n = 10)	P value
Age (years) ± mean (SD)	59.08 ± 10.65^*^	68.46 ± 12.32	64.30 ± 15.64	<0.001
Sex (male), n (%)	83 (79.8%)	33 (66.0%)	8 (80.0%)	0.165
Hypertension	66 (63.5%)	39 (78.0%)	7 (70.0%)	0.191
Diabetes	28 (26.9%)	20 (40.0%)	3 (30.0%)	0.259
Coronary Heart Disease	10 (9.6%)	6 (12.0%)	2 (20.0%)	0.582
Atrial Fibrillation	16 (15.4%)^*^	16 (32.0%)	1 (10.0%)	0.039
Hyperlipidemia	35 (33.7%)	8 (16.0%)	2 (20.0%)	0.061
Previous Stroke, n (%)	23 (22.1%)	15 (30.0%)	5 (50.0%)	0.123
Smoking status, n (%)	64 (61.5%)	26 (52.0%)	6 (60.0%)	0.529
Drinking status, n (%)	50 (44.2%)	15 (30.0%)	4 (40.0%)	0.266
NIHSS on Admission, median (IQR)	6 (3,10)^*#^	16 (8,23)	21.5 (14.75-26)	<0.001
mRS on Admission, median (IQR)	4 (3,4)^*#^	5 (4,5)	5 (4.75,5)	<0.001
GCS on Admission, median (IQR)	15 (13,15)^*#^	11.5 (7,14)	8 (5.5,10.75)	<0.001
Dysphagia, n (%)	22 (21.2%)^*#^	32 (64.0%)^&^	10 (100%)	<0.001
Enteral Nutrition, n (%)	29 (27.9%)^*#^	47 (94.0%)	10 (100%)	<0.001
MV before pneumonia, n (%)		0 (0.0%)	10 (100%)	
MV after pneumonia, n (%)		42 (84.0%)	10 (100%)	
Oral hygiene care, n (%)	104 (100%)	50 (100%)	10 (100%)	1
Revascularization therapies, n (%)				0.106
Intravenous Thrombolysis	16 (15.4%)	1 (2.0%)	0 (0.0%)	
Endovascular Thrombectom	53 (51.0%)	33 (66.0%)	7 (70.0%)	
Bridging Therapy	11 (10.6%)	7 (14.0%)	2 (20.0%)	
Drug Treatment	24 (23.1%)	9 (18.0%)	1 (10.0%)	
Stroke subtype, n (%)				0.569
Large artery disease	65 (62.5%)	34 (68.0%)	8 (80.0%)	
Cardioembolism	21 (20.2%)	11 (22.0%)	1 (10.0%)	
Small vessel occlusion	10 (9.6%)	1 (2.0%)	0 (0.0%)	
Others	8 (7.7%)	4 (8.0%)	1 (10.0%)	
ICU length of stay, median (IQR)	2 (1,3)^*#^	7.5 (3,15)	11 (5.75,17.5)	<0.001
30-day mRS, median (IQR)	2 (1,3)^*#^	5 (4,5)	5 (4.75,6)	<0.001
90-day mRS, median (IQR)	1 (0,2)^*#^	5 (3,6)	5 (4.75,6)	<0.001
30-day motality, n (%)	4 (3.8%)^*#^	7 (14.0%)	3 (30.0%)	0.005

*SD* standard deviation; *IQR* interquartile range; *SAP* stroke-associated pneumonia; *VAP* ventilator-associated pneumonia; *GCS* Glasgow Coma Scale; *NIHSS* National Institutes of Health Stroke Scale; *mRS* modified Rankin Scale; *MV* Mechanical ventilation. ^*^ SAP compared with nonpneumonia, ^#^ VAP compared with nonpneumonia, ^&^ SAP compared with VAP

**Table 2:** Dental and oral health of patients among the three groups

	nonpneumonia(n = 104)	SAP (n = 50)	VAP (n = 10)	P value
Serious caries, n (%)	63(60.6%)	26(52.0%)	7(70.0%)	0.449
Serious periodontitis, n (%)	53(51.0%)	25(50.0%)	6(60.0%)	0.843
Missing teeth, n (%)	17(16.3%)	7(14.0%)	1(10.0%)	0.831
Residual root, n (%)	27(26.0%)	9(18.0%)	1(10.0%)	0.335
Serious calculus, n (%)	75(72.1%)	32(66.0%)	8(80.0%)	0.591

### Differences in the oral microbiota profile differentiate the SAP, VAP and nonpneumonia groups

**Fig. 2:**
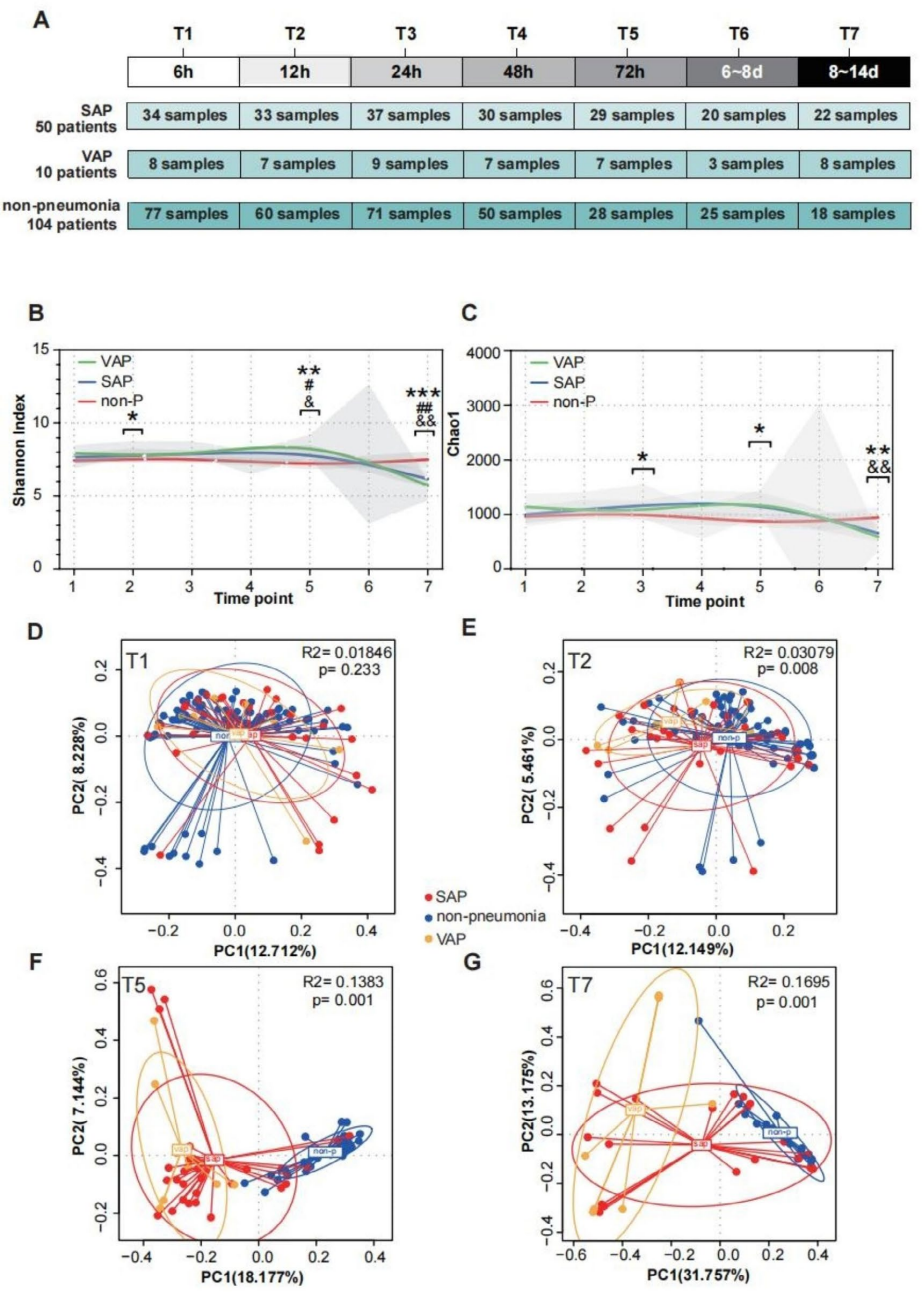
Compositional differences in the oral microbiota of individuals among the three groups at different time points after stroke. (**A**) Trial profile of cohort. The α diversity (Shannon index) (**B**) and richness (Chao1 index) (**C**) at different time points after stroke. **D, E, F, G** PCoA plots based on Bray‒Curtis distances are drawn to display the dynamic differences among the three groups at T1, T2, T5 and T7. Each point represents the composition of the oral microbiota of one participant. P values comparing the different groups were calculated using Wilcoxon tests, *p ≤ 0.05; **p ≤ 0.01; ***p ≤ 0.001;*among three group,# SAP compared with non,& VAP compared with non,%SAP compared with VAP. *SAP* stroke-associated pneumonia; *VAP* ventilator-associated pneumonia; *PCoA* principal coordinate analysis

To determine whether the oral microbiome structures differed significantly among the three groups of AIS patients (164 in total), we collected oral samples at various time intervals following admission. These intervals included 6 h (T1), 12 h (T2), 24 h (T3), 48 h (T4), 72 h (T5), 6 to 8 days (T6), and 8 to 14 days (T7) (Fig. 2A). The α-diversity metrics of the oral microbiota differed significantly over time after stroke (Fig. 2B, C). The microbial diversity and species richness in the SAP and VAP groups were significantly higher from T2 to T5 and then lower at T7 than those in the nonpneumonia group. The PCoA plots based on Bray-Curtis distances showed a temporal shift: there were significant differences in microbiome composition among the three groups at T2 that continued to T7 (Fig. 2D-G, Figure S1). The composition in the SAP and VAP groups showed considerable longitudinal dynamic changes. However, the nonpneumonia patients did not exhibit this characteristic change (Figure S2 A-C). Then, we analyzed the coefficient of variation (CV) of a longitudinal collection of α and β diversity values. We found significant differences in the stability values of the oral microbiota over time between the nonpneumonia and SAP groups. The SAP patients had a high CV, reflecting more variation. Nonpneumonia patients had relatively stable species diversity and community structure after stroke over time (Figure S2 D, E).

### Dynamic characteristics of and trends in the oral microbiota after stroke among the three groups

**Fig. 3:**
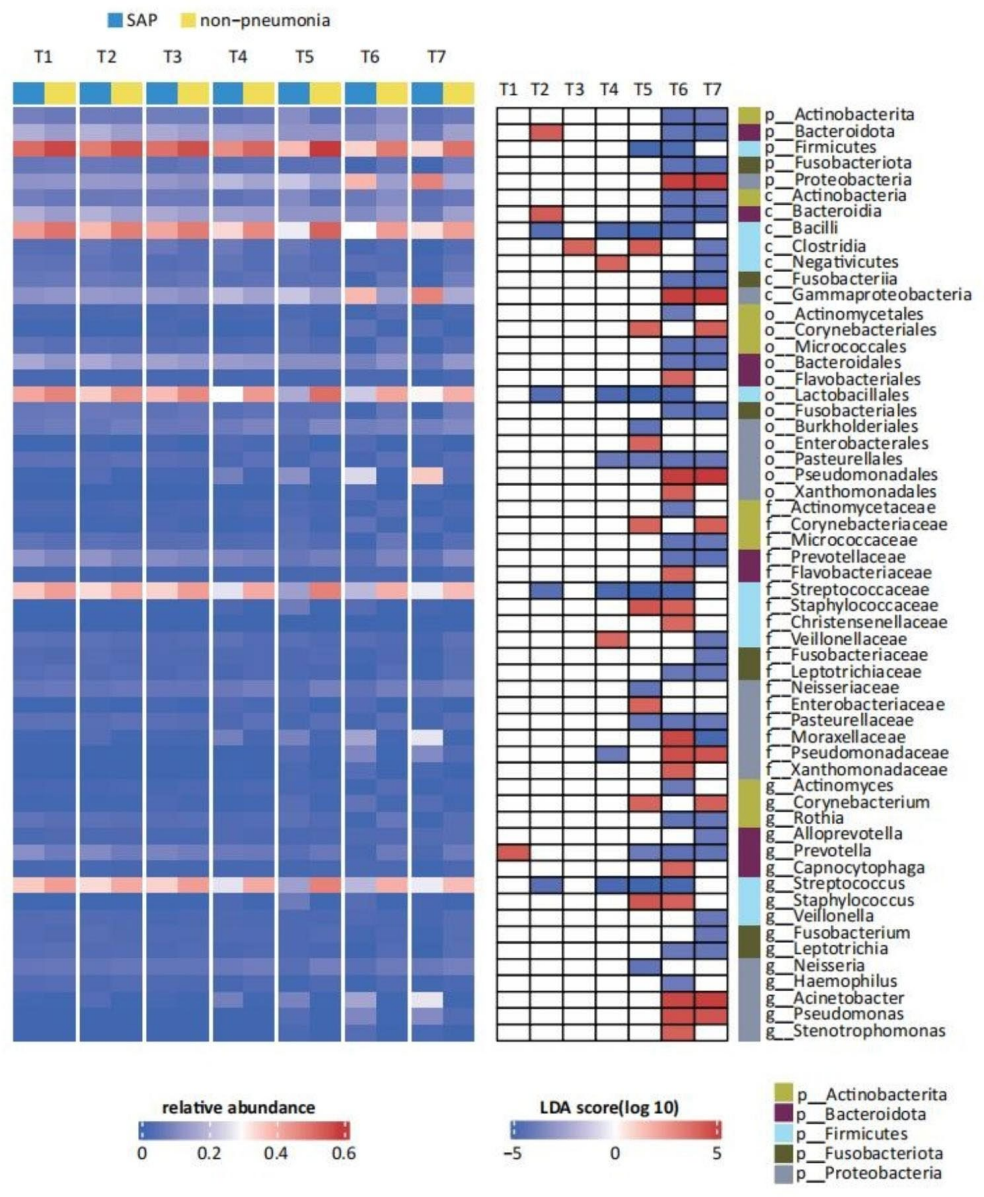
The LEfSe analysis shows that the representation of the various bacterial taxa changed over the time points between SAP and nonpneumonia. Only taxa with a statistically significant LDA score (log10) > 4 are shown. The heatmap on the left shows the relative abundance of the taxa, and the heatmap on the right shows the LDA scores. LEfSe Linear discriminant analysis effect size. p phylum, c class, o order, f family, g genus

To further observe the dynamic changes of oral microbiota after stroke at the taxonomic level, we observed dysbiosis in the oral microbiota profile at the phylum and genus levels at different time points (Figure S3 A-C). We compared the oral microbiota composition at each time point in SAP patients with that at the same time points in the nonpneumonia group through LEfSe. We discovered that 16 genera were significantly altered in SAP participants. The relative abundance of taxa that differed between different time points revealed that the core oral microbes [31–33] in healthy individuals, including the genus *Streptococcus*, was significantly richer in the nonpneumonia group than in the SAP and Pre-SAP groups (Fig. 3, S6). The SAP group was characterized by the enrichment of several pathogens and opportunistic pathogens from T5 to T7, while some bacteria had different change trends over time. The relative abundance of the genera *Acinetobacter* and *Corynebacterium* increased at T5; the abundance of the genera *Staphylococcus* and *Pseudomonas* increased at T6 and T7, respectively (Fig. 3). Moreover, the VAP group showed higher levels of the phylum *Proteobacteria*, the class *Gammaproteobacteria*, the family *Enterobacteriaceae* and the genus *Klebsiella* than the nonpneumonia or SAP group from T4 to T7 (Figure S4, S5).

### Dynamic alterations in microbiome phenotype prediction for oral microbiota in SAP patients

**Fig. 4:**
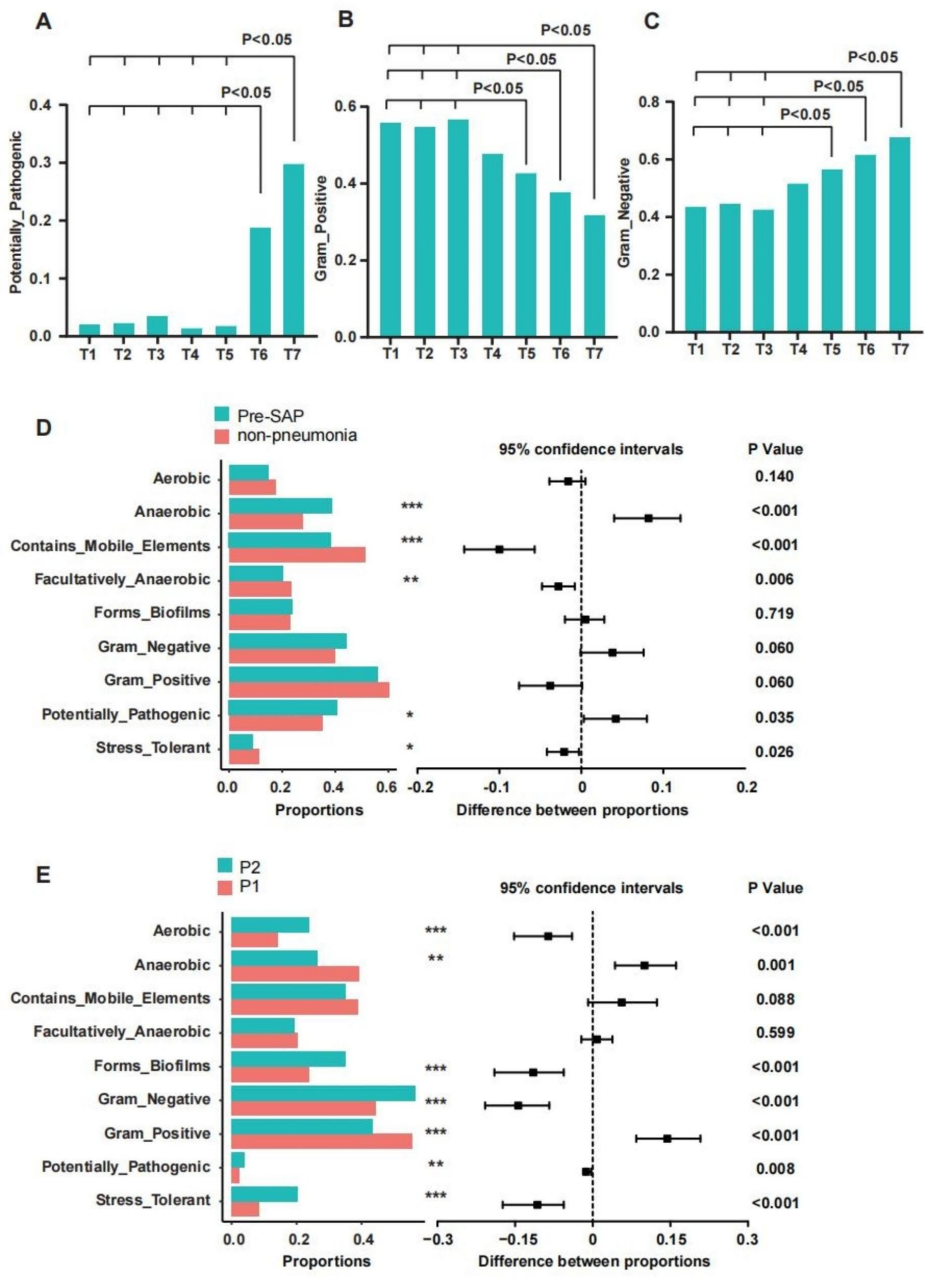
Microbial phenotype prediction by BugBase. (**A,B,C**) Dynamic characteristics of oral microbiota in SAP. The connection of fold lines represents a significant difference between different time points. (**D**) The different microbial compositions between pre-SAP and nonpneumonia. *Pre-SAP* before diagnosis of SAP samples. (**E**) The different microbial compositions between P1 and P2. *P1* before diagnosis of SAP samples; *P2* after diagnosis of SAP samples

To predict microbiome phenotypes within complex microbiomes, we conducted the BugBase. First, when comparing patients with SAP and nonpneumonia, we observed a significant increase in potentially pathogenic bacteria and bacteria associated with biofilm formation in SAP patients (Figure S7). Then, in patients with SAP, we found a significantly higher presence of potentially pathogenic bacteria and bacteria tolerant to oxidative stress, especially between T6 and T7, highlighting the dynamic feature of the oral microbiota in SAP. Additionally, during the T5-T7 period, the representation of Gram-positive and Gram-negative bacteria exhibited contrasting trends (Fig. 4A-C,S8). We were concerned about the functional changes of oral microbiota before the onset of pneumonia, and we have found that potentially pathogenic bacteria and oxidative stress-tolerant bacteria have significantly enriched in SAP before pneumonia compared to nonpneumonia patients (Fig. 4D). Moreover, when comparing the oral microbiota before and after the occurrence of pneumonia, the results indicated an increase in aerobic bacteria, Gram-negative bacteria, potentially pathogenic bacteria, bacteria associated with biofilm formation, and oxidative stress-tolerant bacteria after the diagnosis of SAP (Fig. 4E).

### Microbial dynamic variation may be linked to pneumonia and different invasive and pharmacological medical treatments in SAP

The oral microbiota exhibited profound alterations in diversity and composition over time after stroke in the SAP groups, suggesting that the dynamics of the features of the oral microbiota can potentially respond to host development of pneumonia. Moreover, whether the composition of the oral microbiota is perturbed by invasive and pharmacological medical treatments was unknown.

**Fig. 5:**
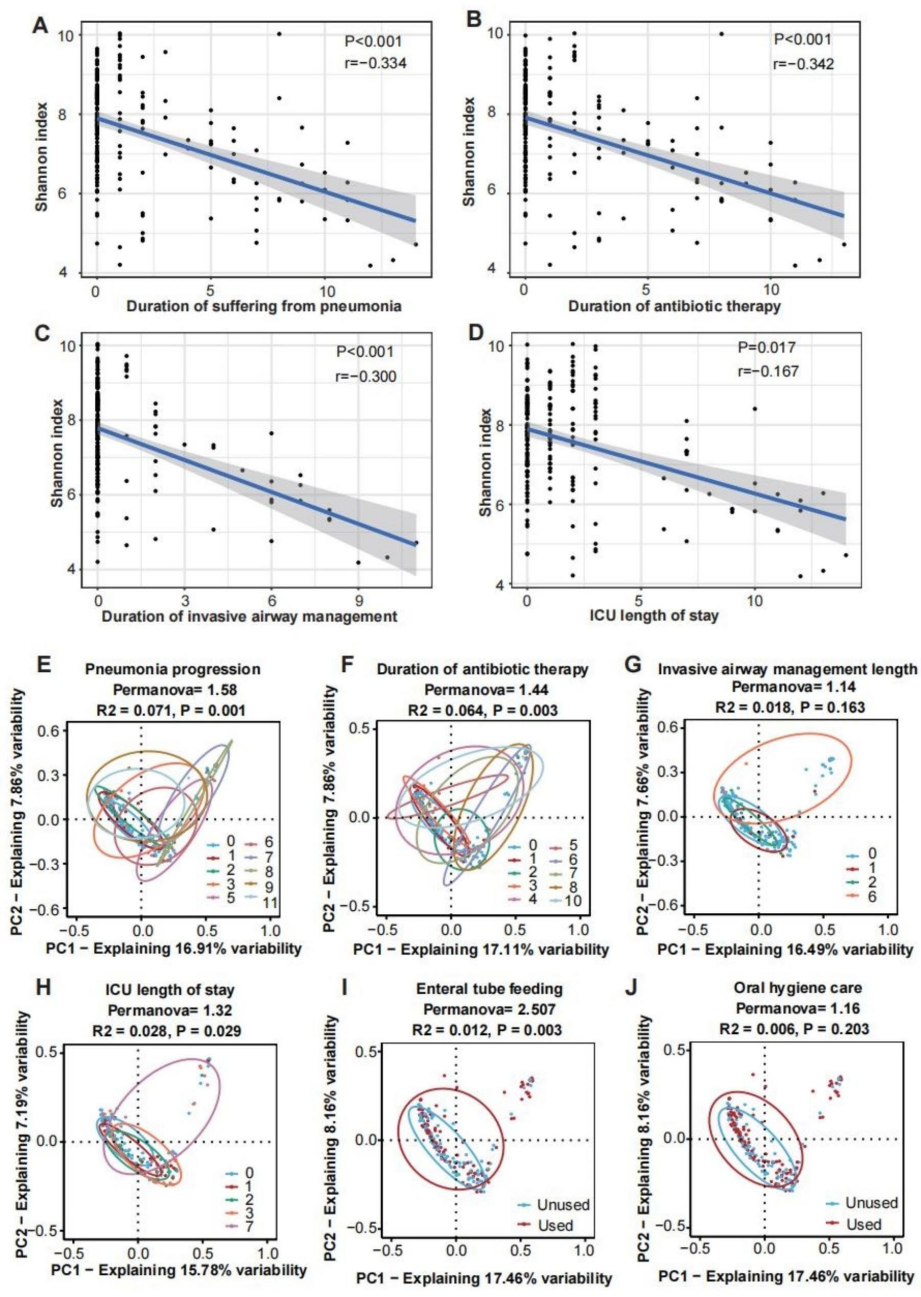
Invasive and pharmacological medical treatments influence the oral microbiota after stroke. The duration of pneumonia (**A**), duration of antibiotic therapy (**B**), duration of invasive airway management (**C**) and ICU length of stay (**D**) were negatively associated with the Shannon index. PCoA results of multidimensional data are shown to display changes in microbial communities according to major variables in SAP: pneumonia progression (**E**), duration of antibiotic therapy (**F**), invasive airway management length of use (**G**), ICU length of stay (H), enteral tube feeding (**I**) and oral hygiene care (**J**). The x- and y-axes represent the two most informative PCs of the PCoA, and marginal boxplots describe the distribution of those values for the different groups. Color legends represent the respective variables under analysis. The number refers to the number of days in **E, F, G, and H**. The results of the PERMANOVA to compare dissimilarity indexes among samples are shown on top of plots accordingly. *PCs* principal coordinates; *PERMANOVA* permutation-based test

First, we found that the α-diversities of the samples from SAP groups, which included only samples collected more than once from a single individual, were contrarily related with the number of days patients suffered from pneumonia (r=-0.334, P < 0.001), antibiotic use (r=-0.342, P < 0.001), the use of invasive airway management (r=-0.300, P < 0.001) and ICU stay duration (r=-0.167, P = 0.017) (Fig. 5A-D, Figure S9).

Permutational multivariate analysis of variance (PERMANOVA) indicated that pneumonia progression could drive significant changes in the microbial community structure (PERMANOVA = 1.58, p = 0.001) in the SAP group (Fig. 5E). However, we discovered that variables of medical treatments could modulate the microbiota as well. As a result, the duration of antibiotic therapy appeared to have a greater impact on the microbiota profiles than other parameters (PERMANOVA = 2.51, p = 0.003 and PERMANOVA = 1.32, p = 0.018 for enteral tube feeding and ICU length of stay, respectively), which was responsible for 6% of the variation in oral microbiota (PERMANOVA = 1.44, p = 0.003 for antibiotic therapy) (Fig. 5F, H, I). However, the invasive airway management duration (PERMANOVA = 1.14, p = 0.163) and oral hygiene care (PERMANOVA = 1.16, p = 0.203) may play a minor role in driving changes in composition in SAP patients (Fig. 5G, J).

Furthermore, through the mixed linear model (LMM), some specific features were identified, indicating that ASVs were strictly associated with each variable. After adjusting the model, the relative abundance of *Fusobacteriota, Lactobacillales, Rothia, Prevotella* and *Streptococcus* were negatively correlated with antibiotic use, and the relative abundance of *Prevotella, Bacteroidota, Rothia, Veillonellaceae* and *Pseudomonas* were negatively correlated with invasive airway management (Table S1).

### Identification of the relative abundance of specific taxa associated with SAP

**Fig. 6:**
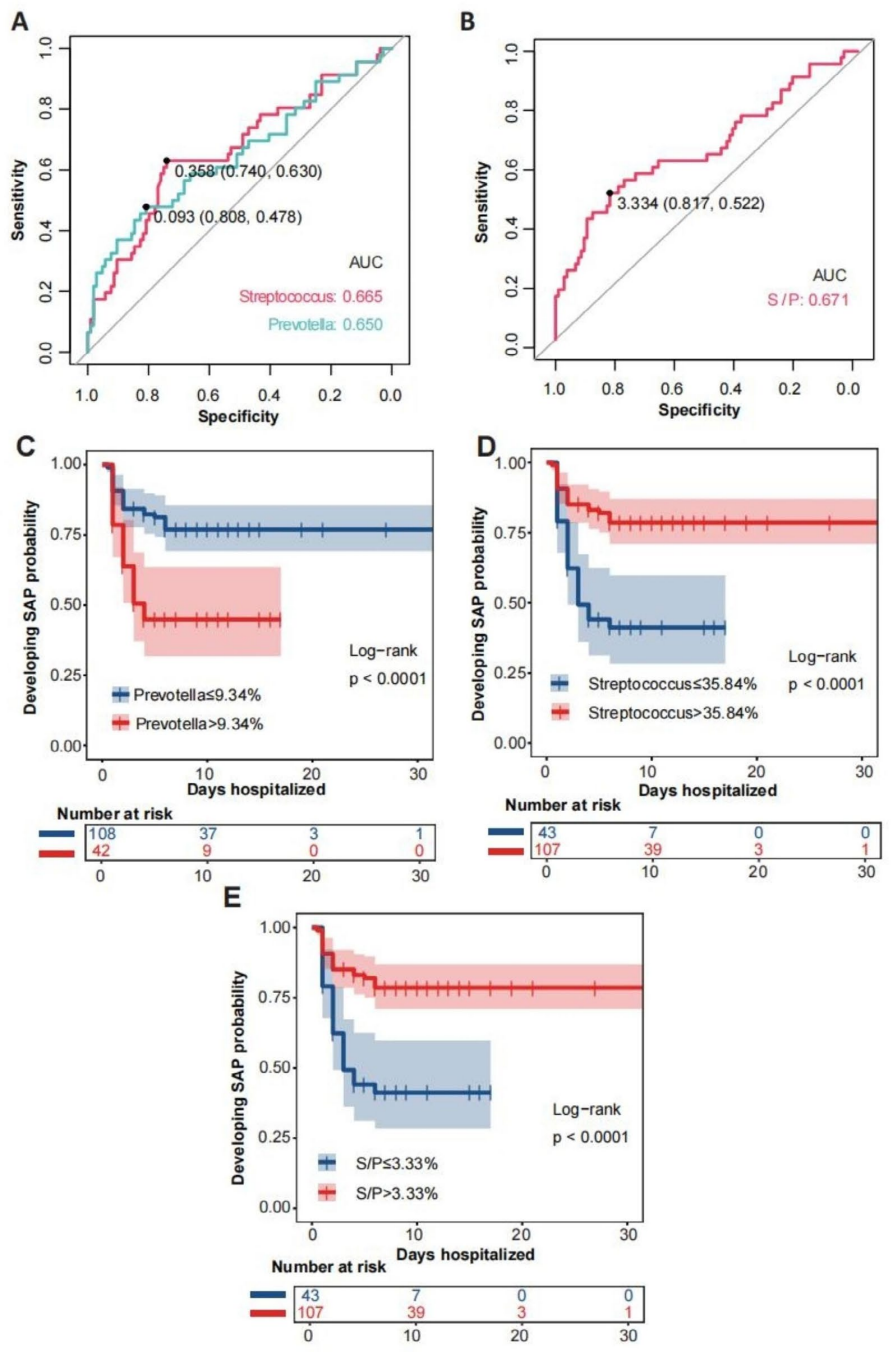
The relative abundance of specific taxa associated with SAP. ROC curve analyses for derivation of thresholds of protective bacterial relative abundance (*Streptococcus*)(**A**), hazardous bacterial relative abundance (*Prevotella*)(**A**) and *Streptococcus/Prevotella* (*S/P*) ratio(**B**). Kaplan‒Meier curves for developing SAP probability during hospitalization stratified by thresholds of protective bacterial relative abundance (*Streptococcus*)(**D**), hazardous bacterial relative abundance (*Prevotella*)(**C**) and *Streptococcus/Prevotella* (*S/P*) ratio(**E**). *P* values were derived from the log-rank test. *ROC* Receiver operator characteristic

We evaluated 46 SAP with 114 longitudinal samples before diagnosis of SAP, as well as 104 nonpneumonia individuals, to determine whether any specific taxa were linked to the risk of developing pneumonia. By utilizing LMM analysis, we identified 11 ASVs significantly associated with SAP. After nonparametric testing, we finally determined that 5 ASVs were inversely correlated with SAP and were considered protective taxa, while there were 5 hazardous ASVs that were positively associated with SAP (Table S3). We sought to develop a simple predictive or hazardous index from these features. Using ROC curves to distinguish the SAP patients, we identified optimal thresholds of protective bacterial relative abundance (35.84% for *Streptococcus*) and hazardous bacterial relative abundance (9.34% for *Prevotella*) (Fig. 6A). Moreover, we determined thresholds of a *Streptococcus*/*Prevotella* (S/P) ratio < 3.33 that distinguished SAP patients from the rest of the cohort (Fig. 6B). Then, to further investigate the impact of the microbial index on SAP, proportions and time-to-event analyses were performed, we found that patients with lower protective bacteria relative abundance (Streptococcus < 35.84%) or S/P ratio(< 3.33)were more likely to suffer from SAP at 10 days after sampling (Fig. 6C-E). We then generated a multivariable Cox proportional hazards model, the log-transformed average of *Streptococcus* (aHR = 0.223, 95% CI 0.063–0.794, p = 0.021; aHR = 0.184, 95% CI 0.056–0.602, p = 0.005) was significantly associated with SAP (Table 3).

**Table 3:** Cox regression analysis of risk factors associated with SAP and the relative abundance of specific taxa

Risk factor	Univariate analysis			Multivariate analysis*			Multivariate analysis^#^	
	HR(95% CI)	P value		Model 1 aHR(95% CI)	P value		Model 2 aHR(95% CI)	P value
**Protective bacteria**								
***p__Firmicutes***	0.016(0.002–0.160)	<0.001		NA	NA		NA	NA
***c__Bacilli***	0.090(0.028–0.290)	<0.001		0.190(0.042–0.864)	0.032		0.139(0.035–0.552)	0.005
***o__Lactobacillales***	0.095(0.031–0.292)	<0.001		0.183(0.044–0.766)	0.020		0.154(0.043–0.557)	0.004
***f__Streptococcaceae***	0.125(0.043–0.367)	<0.001		0.223(0.063–0.794)	0.021		0.184(0.056–0.602)	0.005
***g__Streptococcus***	0.125(0.043–0.367)	<0.001		0.223(0.063–0.794)	0.021		0.184(0.056–0.602)	0.005
**Hazardous bacteria**								
***p__Bacteroidota***	4.732(1.434–15.618)	0.011		NA	NA		NA	NA
***c__Bacteroidia***	4.735(1.435–15.636)	0.011		NA	NA		NA	NA
***o__Bacteroidales***	4.557(1.492–13.913)	0.008		NA	NA		NA	NA
***f__Prevotellaceae***	4.147(1.559–11.032)	0.004		NA	NA		NA	NA
***g__Prevotella***	4.508(1.781–11.778)	0.002		NA	NA		3.224(1.147–9.063)	0.026
** F/B**	0.254(0.107–0.601)	0.002		NA	NA		NA	NA
**S/P**	0.345(0.202–0.590)	<0.001		NA	NA		0.424(0.231–0.776)	0.005

*adjusted by age, initial NIHSS score, enteral nutrition, atrial fibrillation, dysphagia, and NLR(neutrophil to lymphocyte ratio)

#adjusted by A2DS2 score

*F/B* Firmicute/Bacteroidota (F/B) ratio; *S/P* Streptococcus/Prevotella (S/P) ratio

### Associations between dysbiosis of the oral microbiota and clinical outcomes

SAP patients had a worse clinical prognosis after stroke. To investigate the potential association between oral microbiota and poor clinical prognosis, we evaluated 50 SAP patients and collected a total of 205 longitudinal samples (before diagnosis of SAP (P1) = 101 samples and after diagnosis of SAP (P2) = 104 samples). First, we attempted to identify a specific oral microbiota signature through LMM analysis associated with clinical outcomes and mortality by removing the potential effect of all mentioned latent confounding variables (Table S5).

Then, logistic regression was used to generate a potential predictive model of clinical outcomes in SAP patients based on changes associated with the abundance of ASVs calculated by LMM analysis. After adjusting for indicators of clinical indexs, the log-transformed average abundance of *Actinobacteriota* (P1), *Actinobacteria* (P1), *Actinomycetales* (P1) and *Actinomycetaceae* (P1) was significant in both the univariate and multivariate analyses, indicating that certain taxa in the phylum of the *Actinobacteriota* enrichment remained independent risk factors for 30-day functional poor outcomes (Table 4). Regarding 90-day functional outcomes, the log-transformed average abundance of *Actinobacteriota* (P1) was found to be significantly associated in the univariate and multivariate logistic regression analyses (Table S7).

**Table 4:** Logistic regression analysis of risk factors associated with poor 30-day outcomes in SAP patients

Risk factor	Univariate analysis				Multivariate analysis*			Multivariate analysis^#^	
	OR(95% CI)	P value			Model 1 OR(95% CI)	P value		Model 2 OR(95% CI)	P value
**P1**									
***p__Actinobacteriota***	25.837(1.367-488.456)		0.030		22.954(1.376-383.026)	0.029		24.270(1.338-440.331)	0.031
***c__Actinobacteria***	11.632(1.163-116.352)		0.037		12.932(1.316-127.104)	0.028		13.061(1.178-144.754)	0.036
***o__Actinomycetales***	50.309(1.881-1345.855)		0.019		22.372(1.075-465.609)	0.045		63.506(1.643-2454.396)	0.026
***f__Actinomycetaceae***	51.187(2.054-1275.877)		0.016		23.662(1.190-470.657)	0.038		62.317(1.721-2256.818)	0.024
***g__Actinomyces***	5.162(1.049–25.411)		0.044		NA	NA		NA	NA
**P2**									
***p__Actinobacteriota***	9.773(1.230-77.633)		0.031		NA	NA		119.502(1.283-11133.762)	0.039
***c__Actinobacteria***	5.845(1.013–33.727)		0.048		NA	NA		NA	NA
**Δ**									
***f__Actinomycetaceae***	0.148(0.023–0.967)		0.046		NA	NA		0.043(0.002–0.865)	0.040
***g__Actinomyces***	0.143(0.021–0.987)		0.048		NA	NA		0.055(0.003–0.974)	0.048

* Model 1 adjusted by SOFA, APACHE II, initial GCS score and length of ICU stay

# Model 2 adjusted by age, initial NIHSS score, enteral nutrition, atrial fibrillation, dysphagia, and NLR (neutrophil to lymphocyte ratio)

P1 = log-transformed average (before diagnosis of SAP samples); P2 = log-transformed average (after diagnosis of SAP samples); Δ = P2-P1.

**Fig. 7:**
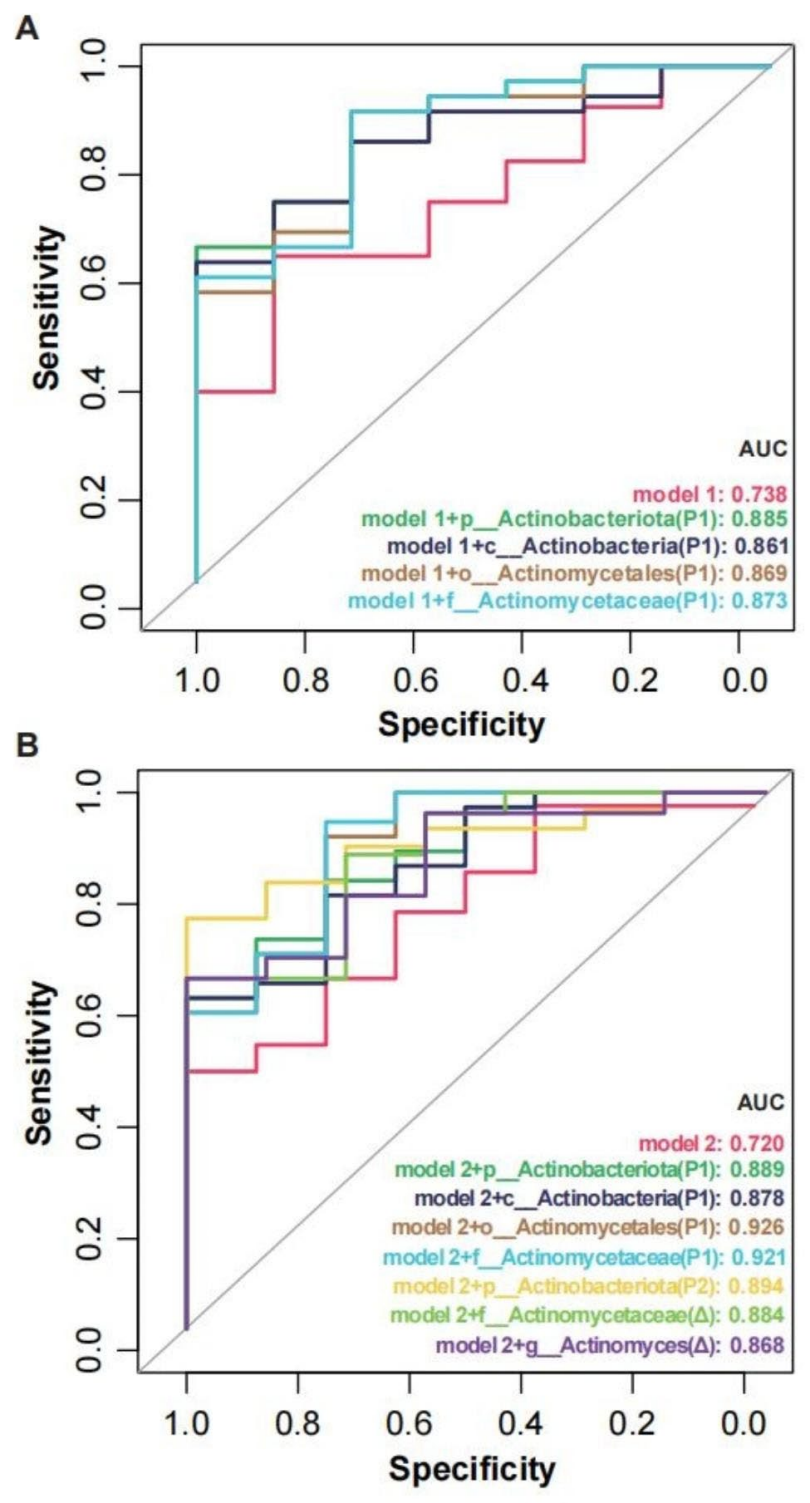
Predictive models based on microbiota and clinical index for early distinction of 30-day poor outcomes. Poor (mRS ≥ 3) and good (mRS<3) outcomes at the 30-day follow-up were defined in patients. The candidate variables of microbiota with an adjusted p value of < 0.05 in the multivariate logistic regression model. Model 1 (**A**) included age, initial NIHSS score, enteral nutrition, atrial fibrillation, dysphagia, and NLR. Model 2 (**B**) included indicators of critical illness (GCS, APACHE-II and SOFA scores and the length of ICU stay). *mRS* modified Rankin Scale; *NIHSS* National Institutes of Health Stroke Scale; *NLR* neutrophil to lymphocyte ratio; *GCS* Glasgow Coma Scale; *APACHE-II* Acute Physiology and Chronic Health Evaluation; *SOFA* Sequential Organ Failure Assessment scores

To assess the potential use of the oral microbiota as a biomarker for early prediction of 30-day functional outcomes, ROC curve analysis was performed. We found that the predictive performance could be improved significantly by combining the clinical index model with the associated taxa in the phylum of the *Actinobacteriota* identified in logistic regression analysis (Fig. 7A, B).

## Discussion

In this prospective observational study of AIS patients, it was observed that patients with SAP and VAP experienced longer hospital stays and had more unfavorable outcomes. Additionally, the comparative taxonomic profiles of the SAP and VAP groups demonstrated a significant microbial shift at various timepoints following stroke. Furthermore, the oral microbial composition during the early acute stage in these groups differed from that of nonpneumonia patients. To predict SAP at an early stage, we conducted a study on the relationship between pathogenic bacteria and adverse outcomes, as well as the protective properties of bacteria derived from the oral cavity. Our analysis using Cox regression revealed that Streptococcus exhibited a protective effect against SAP. These findings provide valuable insights into the oral microbiota ecology in stroke patients and pivotal predictive factors in microbial profiles. Additionally, logistic regression analyses showed that enrichment of certain taxa in the phylum *Actinobacteriota* was an independent risk factor for poor 30-day clinical outcomes in the SAP group.

The oral microbiota is the second most diverse microbial community that colonizes the human body, following the intestinal microbiota [34]. The role of these microbes in maintaining oral and systemic health is crucial. When the oral microbiota becomes dysbiotic, it can impact the development of various diseases. Therefore, this community has been suggested as a potential source of valuable disease biomarkers [35–43]. Existing evidence clearly indicates that poor oral health and dysbiosis of the oral microbiota contribute to healthcare-associated infections and impact the deterioration of clinical outcomes [18, 19, 44, 45]. In recent times, substantial evidence has emerged suggesting that respiratory tract dysbiosis could be a crucial factor contributing to variations in systemic inflammatory responses [46] and clinical outcomes at the patient level during critical illness. Furthermore, it has been found that this dysbiosis can be altered [20, 47–49]. Consistent with the findings of prior studies [16, 17], we have confirmed that the microbiome in patients with SAP/VAP significantly differs from that in nonpneumonia AIS participants. This distinction could potentially have implications for clinical outcomes. To confirm the relationship between this microbiota imbalance and the onset of pneumonia, we have discovered that, apart from the significant impact pneumonia has on the microbiota of stroke patients, various interventions such as antibiotic treatment, invasive airway manipulation, oral hygiene maintenance, and time spent in the ICU can also influence the microbial composition. Therefore, it is probable that this dysbiosis occurs due to the stress caused by factors related to illness, invasive treatments, and pharmacological interventions.

Our findings that protective bacterial features, such as *Streptococcus* abundance and the *S/P* ratio, were significantly associated with SAP indicated that stroke patients with a lower relative abundance of protective bacteria were more likely to suffer from SAP. *Streptococcus* [50], *Neisseria*, *Prevotella* [51], *Rothia*, *Haemophilus*, and *Fusobacterium* were recently described to form the core oral microbiota in healthy individuals [31–33]. In line with previous reports [16, 17], *Streptococcus* were considered normal residents of the oral cavity in stroke patients. Participants who had a high prevalence of Streptococcus in their oral cavity tended to possess a more varied microbiota and a lower occurrence of systemic infections, indicating a potential protective effect [50, 52–54]. For example, *Streptococcus salivarius* is believed to function as a probiotic by producing bacteriocins that hinder the growth of other bacteria and support a balanced oral environment [55–57]. On the other hand, *Streptococcus pneumoniae* [58, 59] and *Streptococcus pseudopneumoniae* [60] appear to be respiratory tract colonizers with the potential to become pathogenic. Our findings provide evidence that maintaining a healthy balance in the core oral microbiota is crucial in reducing the risk of pneumonia and other infections, rather than the presence or absence of any single species or group of bacteria. However, due to the limited precision of the 16 S rRNA gene tag and the relatively small sample size, the results of our analysis are insufficient to identify specific protective species within the *Streptococcus* group. Nevertheless, the link between oral *Streptococcus* symbiosis and pneumonia prognosis could help to identify potential targets for predicting SAP in the future.

Moreover, our results showed that the increases in the abundance of *Actinobacteriota*, *Actinomycetales*, *Actinomycetaceae* and *Actinomyces* were associated with poor outcomes within 30 days in the SAP group. *Actinomyces* are colonizers of oral cavities in humans and are involved in the development of respiratory tract infections, which has been well documented [61, 62]. Notably, *Actinomyces* are recognized as significant sources of antibiotic resistance [63, 64], which may be attributed to the presence of patients with infectious diseases and frequent exacerbations, making them important microbial reservoirs. As a result, long-term antibiotic regimens were recommended to minimize bacterial burden and inflammation. However, the utilization of this strategy creates an environment that promotes antimicrobial resistance due to selective pressure [65, 66], which is a major global concern and serious threat to public health [67]. In addition, disruption of the oral microbiome may affect central nervous system (CNS) functions [54, 68–70]. *Actinomyces* has been identified in recent research as being connected to brain dysfunction or infection [71–73], and oral exposure to *Actinomyces meyeri* has led to an increase in the production of β-amyloid 42 protein and macrophage infiltration in mouse brains [74]. As a result, we believe that *Actinomyces* is a distinct type of oral bacterium with a correlation to both the functioning of the central nervous system and clinical outcomes.

As the oral cavity is hypothesized to play key roles in the progression of SAP or VAP, the restoration of commensal “healthy microbes” or the eradication of pathogens used to be considered beneficial effects in patients. Microbiome associated intervention therapy includes antibiotic treatment, probiotic administration [75–77] and oral hygiene care [14, 22–24, 78]. However, our results indicated that antibiotic therapy was inversely correlated with the healthy core oral microbiota (including *Streptococcus*, *Prevotella*, *Rothia* and *Fusobacterium*). This finding is consistent with previous research [17]. Although antibiotics play a vital role in managing stroke-related infections, there are still challenges and uncertainties. The existing guidelines do not recommend the use of prophylactic antibiotic treatment (PAT) before confirming a diagnosis of SAP [79]. The effectiveness of PAT in preventing infection and enhancing prognosis is still a contentious topic, and currently, no beneficial effects of antibiotics in preventing SAP have been identified.

It has been proven that oral diseases are closely linked to the occurrence of pneumonia [80, 81]. However, in our study, there was no difference in oral health among the three groups. On one hand, this may be attributed to the small number of patients included. On the other hand, it could also be due to a lack of professional dental and oral scoring.

The study’s strengths lie in its longitudinal prospective design, speedy participant recruitment within 24 h post-stroke, and meticulous microbial sampling. Nonetheless, there were several limitations to the study that should not be overlooked. Firstly, our findings were restricted to 16 S rRNA gene sequencing, resulting in the inability to analyze viability and virulence factors or attain species-level resolution. Secondly, as it was a single-center study without a control group, external validation was lacking. However, comparing healthy community-dwellers to stroke patients receiving equivalent nursing care was not feasible or comprehensive, resulting in the use of stroke patients without pneumonia as an internal control group due to their similar neurological risk profiles. Thirdly, professional oral cavity assessments and scores were not conducted due to the urgency of treatment for AIS patients admitted within 24 h of stroke onset. Fourthly, the sampling method for oral microbiota is oral swabs, which may be different from previous studies. AIS patients will receive thrombolysis therapy and antiplatelet therapy after admission, considering that collecting dental plaques is an invasive procedure that may cause periodontal bleeding. Moreover, individuals with AIS often experience dysphagia and may be placed on ventilators, which further complicates the task of collecting saliva samples. Therefore, in our study, we opted to gather samples from the buccal mucosa and gingiva instead. Therefore, we ultimately collected samples from buccal mucosa and gingiva. Lastly, the unequal distribution of patients between the groups and the small number of VAP patients were additional limitations. All patients meeting the inclusion criteria were included in the study across a 9-month period, but there were fewer patients with more severe strokes who were intubated during the first seven days after stroke onset in the stroke unit.

## Conclusions

To the best of our knowledge, this prospective observational study is the largest longitudinal cohort study of AIS patients to analyze the oral microbiota using 16 S rRNA gene sequencing. The aim of this study is to describe the oral microbial characteristics before or after pneumonia in the context of AIS. Then, by controlling the potential effect of all latent confounding variables, we assessed the changes associated with pneumonia after stroke and explored the abundances of specific bacteria that were important risk factors for pneumonia and 30-day clinical outcomes in SAP patients.

### Electronic supplementary material

Below is the link to the electronic supplementary material.

Supplementary Material 1

## Data Availability

The raw sequence data reported in this paper have been deposited in the Genome Sequence Archive (Genomics, Proteomics & Bioinformatics 2021) in National Genomics Data Center (Nucleic Acids Res 2022), China National Center for Bioinformation / Beijing Institute of Genomics, Chinese Academy of Sciences (GSA-Human: HRA004928) that are publicly accessible at https://bigd.big.ac.cn/gsa-human/browse/HRA004928.
