# Supplementary material for: Association between oral microbial dysbiosis and poor functional outcomes in stroke-associated pneumonia patients
Source: BMC Microbiol. 2023 Oct 24;23:305. doi: 10.1186/s12866-023-03057-8 (PMC10594709; doi:10.1186/s12866-023-03057-8)
Supplement: Supplementary file 1 — Supplementary Material 1 [file 12866_2023_3057_MOESM1_ESM.docx]

**Supplemental Material**

**Definitions of SAP and VAP and functional outcomes**

SAP following AIS was recorded as the development of lower respiratory tract infections according to the modified Centers for Disease Control and Prevention criteria during the first 7 days after stroke onset[1, 2]. Consensus regarding the criteria for the diagnosis of SAP was reached based on the following: (1) SAP is the recommended terminology for the spectrum of pneumonia complications that occur during the first 7 days after stroke onset in nonventilated patients; (2) modified Centers for Disease Control and Prevention (CDC) criteria are proposed for SAP as follows: probable SAP: CDC criteria met, but typical chest X-ray changes absent even after repeat or serial chest X-ray; definite SAP: CDC criteria met, including typical chest X-ray changes; (3) there is limited evidence for a diagnostic role of white blood cell count or C-reactive protein in SAP; and (4) there is insufficient evidence for the use of other biomarkers (e.g., procalcitonin). The treating physician diagnosed SAP during hospitalization according to the criteria mentioned above. Treating physicians were blinded to the other clinical and laboratory findings regarding the primary diagnosis, as well as the secondary validation. The onset of clinical symptoms led to further investigations and resulted in the diagnosis of SAP.

According to recommendations from the Pneumonia in Stroke Consensus Group, existing diagnostic criteria for VAP include that patients receive mechanical ventilation in the first 7 days after stroke[3-8]. Additional diagnosis criteria were (1) age > 18 years; (2) intubation and mechanical ventilation for > 48 h. Suspected VAP was defined by (1) systemic signs of infection [temperature > 38 or < 36.5°C; white blood cell count < 4000 or > 12,000/mm^3^, purulent tracheal secretions], and (2) new infiltrates on chest X-ray. A confirmed VAP diagnosis required the additional detection of a causative pathogen either by microbiological culture or microbiome analysis from a tracheal sample.

Poor (mRS≥3) and good (mRS＜3) outcomes at the 30- and 90-day follow-ups were defined by telephone.

**Oral hygiene care and dental examinations**

Chlorhexidine (CHX) has been the most commonly used product for oral hygiene. Patients were subjected to oral washing with 15 mL of 2% CHX digluconate by a trained nursery team. The CHX solution was gently brushed into the gum, oral mucosa and tongue two/four times daily until discharge.

All dental examinations were performed bedside by two dentists. Dental Serious caries was recorded at the cavitation stage. Teeth were charted as missing, healthy, decayed, root remnants. We defined the probing pocket depths of >6 mm and marginal alveolar bone loss of >30% as serious periodontitis. Participants had a history of previous calculus formation (> 3 mm of calculus width)were defined as serious calculus.

**16S RNA sequencing and analysis**

DNA was extracted using the Mabio® Bacterial DNA Extraction Mini Kit for oral swab samples. The concentration and purity were measured using a NanoDrop One (Thermo Fisher Scientific, MA, USA). Distinct regions (V3-V4) of the 16S rRNA were amplified using specific primers (338F and 806R) with a 12 bp barcode. Primers were synthesized by Invitrogen (Invitrogen, Carlsbad, CA, USA). PCR samples contained 25 μl 2x Premix Taq (Takara Biotechnology, Dalian Co. Ltd., China), 1 μl each primer (10 mM) and 3 μl DNA (20 ng/μl) template in a volume of 50 µl, and amplification was performed by thermocycling using the following: 5 min at 94°C for initialization; 30 cycles of 30 s denaturation at 94°C, 30 s annealing at 52°C, and 30 s extension at 72°C; followed by 10 min final elongation at 72°C. The PCR instrument was a Bio-Rad S1000 (Bio-Rad Laboratory, CA, USA). The length and concentration of the PCR product were measured by 1% agarose gel electrophoresis. PCR products were mixed in equidensity ratios according to GeneTools Analysis Software (Version 4.03.05.0, SynGene). Then, the mixed PCR products were purified with an E.Z.N.A. Gel Extraction Kit (Omega, USA). Each project involved the appropriate primers for amplification. When the final primer sequence was not known, it could be viewed in the mapping file of the analysis result package. Sequencing libraries were generated using the NEBNext® Ultra™ II DNA Library Prep Kit for Illumina® (New England Biolabs, MA, USA) following the manufacturer's recommendations, and index codes were added. The library quality was assessed on a Qubit@ 2.0 Fluorometer (Thermo Fisher Scientific, MA, USA). Finally, the library was sequenced on an Illumina Nova6000 platform, and 250 bp paired-end reads were generated (Guangdong Magigene Biotechnology Co., Ltd. Guangzhou, China). The Primary FastQ files were partitioned into several files for processing and subjected to quality filtration using Trimmomatic. The subsequent criteria were applied for merging the sequences: (1) If any region within a 50 bp sliding window exhibited an average quality score below 20, the corresponding reads were trimmed. (1) The primers were required to have a perfect match, with the allowance of a maximum of two nucleotide mismatches. Additionally, reads containing ambiguous bases were excluded. (3) Sequences with overlaps exceeding 10 bp were combined by considering their overlapping sequences. The DADA2 method was implemented to detect and correct sequencing noise and remove chimeric sequences. The sequences were grouped into amplicon sequence variants (ASVs) with 99% similarity. To classify the sequences according to their taxonomic information, the q2-feature-classifier plugin was used based on the vsearch alignment method with the SILVA v132 database.

**Statistical analyses**

The Shannon index, Simpson index, and Chao1 index were determined to assess α-diversity. Bray‒CurtisCurtis plots were used to analyze the β-diversity by illustrating the phylogenetic dissimilarity among samples. A smaller distance between two samples indicates a higher similarity. As a dimensionality reduction method, principal coordinate analysis (PCoA) was used to describe the relationships among samples based on the distance matrix and visualize the unsupervised grouping pattern of the microbiome. Because the microbiome data are multidimensional, we used the Adonis test implemented in QIIME 1.9.0 (a method similar to PERMANOVA, which partitions a distance matrix among sources of variation to describe the strength and significance that a categorical or continuous variable has in determining variation in distances). Results with a P value<0.05 were considered statistically significant in the comparisons of groups. Linear discriminant analysis effect size (LEfSe) was used to compare the discriminative data between groups. Significantly different bacteria with LDA scores of ≥ 4.0 were used to generate a taxonomic heatmap. Intrapatient temporal variability of microbial diversity was defined as the coefficient of variation (CV) of a longitudinal collection of α-diversity values and was calculated for each patient’s oral samples. Higher values were indicative of more variable microbial diversity. The temporal variability in community composition, or β-diversity, in each patient was determined in the oral cavity and stool samples by calculating the CV of the Bray‒Curtis distances using longitudinal samples collected from each individual[9]. Based on high-quality sequences, BugBase was used to infer and compare organism-level microbiome phenotypes among different samples[10]. A mixed linear model (LMM) was used to measure the association of log-transformed ASV data with individual variables recorded in the metadata. Idiosyncratic variation due to individual differences was set as a random effect for each variable analyzed (fixed effect). Then, using the ASVs that showed a significant association (p < 0.05), the model was adjusted to control the effects of other variables so that the resulting variation was independent of other variables and not subject to confounding effects from the correlated variables. We classify ASVs that show a significant negative association with SAP occurrence in the LMM analyses as "protective" species. On the other hand, ASVs that show a significant positive association with SAP occurrence are labeled as " hazardous " species. We then utilized ROC curves to define optimal thresholds of the abundances of specific bacteria. After identifying these thresholds, we stratified our cohort by the index and evaluated associations with SAP using Kaplan‒Meier curves. Furthermore, univariate and multivariate Cox proportional hazards models were used to evaluate the association between the abundance of specific taxa and SAP.

1. Hannawi Y, Hannawi B, Rao CP, Suarez JI, Bershad EM: **Stroke-associated pneumonia: major advances and obstacles**. *Cerebrovasc Dis* 2013, **35**(5):430-443.

2. Smith CJ, Kishore AK, Vail A, Chamorro A, Garau J, Hopkins SJ, Di Napoli M, Kalra L, Langhorne P, Montaner J *et al*: **Diagnosis of Stroke-Associated Pneumonia: Recommendations From the Pneumonia in Stroke Consensus Group**. *Stroke* 2015, **46**(8):2335-2340.

3. American Thoracic S, Infectious Diseases Society of A: **Guidelines for the management of adults with hospital-acquired, ventilator-associated, and healthcare-associated pneumonia**. *Am J Respir Crit Care Med* 2005, **171**(4):388-416.

4. Cilloniz C, Torres A, Niederman MS: **Management of pneumonia in critically ill patients**. *BMJ* 2021, **375**:e065871.

5. Metersky ML, Kalil AC: **Management of Ventilator-Associated Pneumonia: Guidelines**. *Clin Chest Med* 2018, **39**(4):797-808.

6. Kalil AC, Metersky ML, Klompas M, Muscedere J, Sweeney DA, Palmer LB, Napolitano LM, O'Grady NP, Bartlett JG, Carratala J *et al*: **Management of Adults With Hospital-acquired and Ventilator-associated Pneumonia: 2016 Clinical Practice Guidelines by the Infectious Diseases Society of America and the American Thoracic Society**. *Clin Infect Dis* 2016, **63**(5):e61-e111.

7. Spalding MC, Cripps MW, Minshall CT: **Ventilator-Associated Pneumonia: New Definitions**. *Crit Care Clin* 2017, **33**(2):277-292.

8. Torres A, Niederman MS, Chastre J, Ewig S, Fernandez-Vandellos P, Hanberger H, Kollef M, Li Bassi G, Luna CM, Martin-Loeches I *et al*: **International ERS/ESICM/ESCMID/ALAT guidelines for the management of hospital-acquired pneumonia and ventilator-associated pneumonia: Guidelines for the management of hospital-acquired pneumonia (HAP)/ventilator-associated pneumonia (VAP) of the European Respiratory Society (ERS), European Society of Intensive Care Medicine (ESICM), European Society of Clinical Microbiology and Infectious Diseases (ESCMID) and Asociacion Latinoamericana del Torax (ALAT)**. *Eur Respir J* 2017, **50**(3).

9. Galloway-Pena JR, Smith DP, Sahasrabhojane P, Wadsworth WD, Fellman BM, Ajami NJ, Shpall EJ, Daver N, Guindani M, Petrosino JF *et al*: **Characterization of oral and gut microbiome temporal variability in hospitalized cancer patients**. *Genome Med* 2017, **9**(1):21.

10. Ward T LJ, Meulemans J, Hillmann B, Lynch J, Sidiropoulos D, Spear JR, Caporaso G, Blekhman R, Knight R.: **BugBase predicts organismlevel microbiome phenotypes.** . *bioRxiv* 2017.

**Supplementary Tables**

**Table S1 Interaction among the significantly discriminative taxa according LefSe and recorded variables in the SAP group.**

| **Variable** | **Groups** | **ASV_S_ associated** | | **Top ASV_s_ associated** | **P Value** | **Adjusted P Value** |
| --- | --- | --- | --- | --- | --- | --- |
| **Antibiotic treatment** | **Yes**/No | 51 |  | *p__Fusobacteriota* | ＜0.001 | ＜0.001(-0.695) |
|  |  |  |  | *c__Fusobacteriia* | ＜0.001 | ＜0.001(-0.695) |
|  |  |  |  | *o__Micrococcales* | ＜0.001 | 0.001(-0.543) |
|  |  |  |  | *o__Lactobacillales* | ＜0.001 | ＜0.001(-0.347) |
|  |  |  |  | *f__Micrococcaceae* | ＜0.001 | 0.001(-0.544) |
|  |  |  |  | *f__Prevotellaceae* | ＜0.001 | ＜0.001(-0.492) |
|  |  |  |  | *f__Streptococcaceae* | ＜0.001 | ＜0.001(-0.442) |
|  |  |  |  | *g__Rothia* | ＜0.001 | 0.001(-0.543) |
|  |  |  |  | *g__Prevotella* | ＜0.001 | ＜0.001(-0.648) |
|  |  |  |  | *g__Streptococcus* | ＜0.001 | ＜0.001(-0.443) |
| **Invasive airway management** | **Yes**/No | 46 |  | *o__Bacteroidales* | ＜0.001 | ＜0.001(-0.447) |
|  |  |  |  | *g__Prevotella* | ＜0.001 | 0.003(-0.391) |
|  |  |  |  | *f__Prevotellaceae* | ＜0.001 | ＜0.001(-0.422) |
|  |  |  |  | *f__Micrococcaceae* | ＜0.001 | ＜0.001(-0.642) |
|  |  |  |  | *p__Bacteroidota* | ＜0.001 | ＜0.001(-0.386) |
|  |  |  |  | *g__Rothia* | ＜0.001 | ＜0.001(-0.640) |
|  |  |  |  | *c__Bacteroidia* | ＜0.001 | ＜0.001(-0.385) |
|  |  |  |  | *o__Micrococcales* | ＜0.001 | ＜0.001(-0.636 |
|  |  |  |  | *f__Veillonellaceae* | ＜0.001 | ＜0.001(-0.399) |
|  |  |  |  | *o__Pseudomonadales* | ＜0.001 | 0.007(0.561) |
| **Enteral nutrition** | **Yes**/No | 45 |  | *g__Prevotella* | ＜0.001 | 0.148(-0.164) |
|  |  |  |  | *o__Lactobacillales* | ＜0.001 | 0.030(-0.176) |
|  |  |  |  | *f__Micrococcaceae* | ＜0.001 | 0.053(-0.309) |
|  |  |  |  | *g__Rothia* | ＜0.001 | 0.053(-0.309) |
|  |  |  |  | *o__Micrococcales* | ＜0.001 | 0.0523(-0.310) |
|  |  |  |  | *f__Streptococcaceae* | ＜0.001 | 0.102(-0.146) |
|  |  |  |  | *g__Streptococcus* | ＜0.001 | 0.103(-0.146) |
|  |  |  |  | *g__Leptotrichia* | ＜0.001 | 0.008(-0.551) |
|  |  |  |  | *f__Leptotrichiaceae* | ＜0.001 | 0.029(-0.433) |
|  |  |  |  | *f__Prevotellaceae* | ＜0.001 | 0.522(-0.069) |
| **Oral hygiene care** | **Yes**/No | 30 |  | *f__Enterobacteriaceae* | ＜0.001 | ＜0.001(0.652) |
|  |  |  |  | *f__Pseudomonadaceae* | ＜0.001 | 0.01(0.604) |
|  |  |  |  | *g__Pseudomonas* | ＜0.001 | 0.01(0.604) |
|  |  |  |  | *o__Enterobacterales* | ＜0.001 | 0.001(0.582) |
|  |  |  |  | *f__Prevotellaceae* | 0.001 | 0.67(-0.041) |
|  |  |  |  | *g__Rothia* | 0.001 | 0.944(-0.01) |
|  |  |  |  | *f__Micrococcaceae* | 0.001 | 0.953(-0.009) |
|  |  |  |  | *o__Micrococcales* | 0.001 | 0.963(-0.007) |
|  |  |  |  | *g__Prevotella* | 0.002 | 0.603(0.054) |
|  |  |  |  | *o__Pseudomonadales* | 0.003 | 0.435(0.124) |

The comparison's reference group is highlighted in bold. The ASVs that are most representative of each variable in the model are shown; The p-value and the meaning of the interaction are shown in parentheses in the final column after all factors have been adjusted for.

**Table S2 Interaction among the significantly discriminative taxa according LefSe and recorded variables in VAP group.**

| **Variable** | **Groups** | **ASV**_S_ **associated** | | **Top ASV_s_ associated** | **P Value** | **Adjusted P Value** |
| --- | --- | --- | --- | --- | --- | --- |
| Antibiotic treatment | **Yes**/No | 35 |  | *f__Streptococcaceae* | ＜0.001 | 0.138(-0.361) |
|  |  |  |  | *g__Streptococcus* | ＜0.001 | 0.138(-0.361) |
|  |  |  |  | *o__Lactobacillales* | 0.002 | 0.282(-0.271) |
|  |  |  |  | *g__Rothia* | 0.002 | 0.301(-0.393) |
|  |  |  |  | *f__Micrococcaceae* | 0.002 | 0.301(-0.393) |
|  |  |  |  | *o__Micrococcales* | 0.002 | 0.298(-0.394) |
|  |  |  |  | *g__Acinetobacter* | 0.003 | 0.299(0.511) |
|  |  |  |  | *f__Xanthomonadaceae* | 0.003 | 0.296(0.616) |
|  |  |  |  | *o__Pseudomonadales* | 0.003 | 0.629(0.193) |
|  |  |  |  | *c__Actinobacteria* | 0.006 | 0.482(-0.167) |
|  |  |  |  |  |  |  |
| Enteral nutrition | **Yes**/No | 47 |  | *c__Gammaproteobacteria* | ＜0.001 | ＜0.001(0.555) |
|  |  |  |  | *p__Proteobacteria* | ＜0.001 | ＜0.001(0.553) |
|  |  |  |  | *f__Streptococcaceae* | ＜0.001 | 0.016(-0.735) |
|  |  |  |  | *g__Streptococcus* | ＜0.001 | 0.016(-0.735) |
|  |  |  |  | *p__Fusobacteriota* | ＜0.001 | ＜0.001(-1.214) |
|  |  |  |  | *c__Fusobacteriia* | ＜0.001 | ＜0.001(-1.214) |
|  |  |  |  | *o__Fusobacteriales* | ＜0.001 | ＜0.001(-1.214) |
|  |  |  |  | *o__Pseudomonadales* | ＜0.001 | ＜0.001(1.112) |
|  |  |  |  | *o__Enterobacterales* | ＜0.001 | ＜0.001(1.253) |
|  |  |  |  | *g__Prevotella* | ＜0.001 | ＜0.001(-1.029) |
|  |  |  |  |  |  |  |
| Oral hygiene care | **Yes**/No | 38 |  | *g__Streptococcus* | ＜0.001 | 0.154(-0.350) |
|  |  |  |  | *c__Gammaproteobacteria* | ＜0.001 | 0.169(0.253) |
|  |  |  |  | *p__Proteobacteria* | ＜0.001 | 0.174(0.250) |
|  |  |  |  | *o__Enterobacterales* | ＜0.001 | 0.151(0.637) |
|  |  |  |  | *g__Acinetobacter* | ＜0.001 | 0.105(0.732) |
|  |  |  |  | *f__Moraxellaceae* | ＜0.001 | 0.106(0.727) |
|  |  |  |  | *o__Pseudomonadales* | ＜0.001 | 0.360(0.360) |
|  |  |  |  | *g__Klebsiella* | 0.001 | 0.172(0.578) |
|  |  |  |  | *o__Lactobacillales* | 0.002 | 0.390(-0.219) |
|  |  |  |  | *f__Leptotrichiaceae* | 0.002 | 0.436(-0.441) |

The comparison's reference group is highlighted in bold. The ASVs that are most representative of each variable in the model are shown; The p-value and the meaning of the interaction are shown in parentheses in the final column after all factors have been adjusted for.

**Table S3 The specific taxa signature associated with pneumonia for stroke patients.**

| **Variable** | **Groups** | **ASVS associated** | **P Value** | **Adjusted P Value** |
| --- | --- | --- | --- | --- |
| **SAP** | **Yes**/No | *f__Streptococcaceae* | <0.001 | <0.001(-0.133) |
|  |  | *g__**Streptococcus* | <0.001 | <0.001(-0.132) |
|  |  | *o__**Lactobacillales* | <0.001 | <0.001(-0.109) |
|  |  | *c__Bacilli* | <0.001 | <0.001(-0.098) |
|  |  | *g__**Prevotella* | 0.005 | 0.005(0.181) |
|  |  | *f__Prevotellaceae* | 0.012 | 0.012(0.158) |
|  |  | *o__Bacteroidales* | 0.016 | 0.015(0.141) |
|  |  | *p__Firmicutes* | 0.020 | 0.020(-0.051) |
|  |  | *p__Bacteroidota* | 0.023 | 0.023(0.124) |
|  |  | *c__Bacteroidia* | 0.023 | 0.023(0.124) |
|  |  |  |  |  |
| **VAP** | **Yes**/No |  |  |  |
|  |  | *o__Lactobacillales* | 0.044 | 0.044(-0.132) |
|  |  | *g__Klebsiella* | 0.032 | 0.022(1.574) |
|  |  | *g__Stenotrophomonas* | 0.044 | 0.044(1.187) |

The comparison's reference group is highlighted in bold. The ASVs that are most representative of each variable in the model are shown; The p-value and the meaning of the interaction are shown in parentheses in the final column after all factors have been adjusted for.

**Table S4 Cox regression analysis of risk factors associated with VAP and the relative abundance of specific taxa.**

| **Risk factor** | **Univariate analysis** | |  | **Multivariate analysis*** | |  | **Multivariate analysis#** | |
| --- | --- | --- | --- | --- | --- | --- | --- | --- |
|  | **HR（95% CI）** | **P value** |  | **Model 1 aHR（95% CI）** | **P value** |  | **Model 2 aHR（95% CI）** | **P value** |
| **Protective bacteria** |  |  |  |  |  |  |  |  |
| ***o__Lactobacillales*** | 0.005（0.000165-0.137） | 0.002 |  | NA | NA |  | NA | NA |
|  |  |  |  |  |  |  |  |  |
| **Hazardous bacteria** |  |  |  |  |  |  |  |  |
| ***g__Klebsiella*** | 1.384(0.855-2.240) | 0.186 |  | NA | NA |  | NA | NA |
| ***g__Stenotrophomonas*** | 1.362(0.875-2.122) | 0.171 |  | NA | NA |  | NA | NA |

*adjusted by age, initial NIHSS score, enteral nutrition, atrial fibrillation, dysphagia, and NLR(neutrophil to lymphocyte ratio)

#adjusted by A2DS2 score

**Table S5 The specific taxa signature associated with clinical outcomes in SAP.**

| **Variable** | **Groups** | **ASV_S_ associated** | **P Value** | **Adjusted P Value** |
| --- | --- | --- | --- | --- |
| **30-day mortality** | **Yes**/No | *p__**Proteobacteria* | 0.047 | 0.046(-0.287) |
| **30-day mRS** | **Poo**r/Good | *p__Actinobacteriota* | 0.003 | 0.003(0.677) |
|  |  | *c__Actinobacteria* | 0.004 | 0.003(0.664) |
|  |  | *o__Actinomycetales* | 0.007 | 0.008(0.683) |
|  |  | *f__Actinomycetaceae* | 0.007 | 0.008(0.683) |
|  |  | *g__Actinomyces* | 0.019 | 0.020(0.634) |
|  |  | *o__Micrococcales* | 0.021 | 0.028(0.586) |
|  |  | *f__Micrococcaceae* | 0.022 | 0.029(0.584) |
|  |  | *g__Rothia* | 0.022 | 0.029(0.584) |
|  |  | *o__Corynebacteriales* | 0.029 | 0.029(0.777) |
|  |  | *g__Corynebacterium* | 0.030 | 0.030(0.775) |
|  |  | *f__Corynebacteriaceae* | 0.030 | 0.030(0.773) |
|  |  | *g__Staphylococcus* | 0.037 | 0.040(-0.588) |
|  |  | *f__Staphylococcaceae* | 0.037 | 0.040(-0.586) |
|  |  | *f__Leptotrichiaceae* | 0.041 | 0.096(0.423) |
| **90-day mRS** | **Poor**/Good | *p__**Actinobacteriota* | 0.023 | 0.012(0.516) |
|  |  | *c__**Actinobacteria* | 0.031 | 0.017(0.499) |

The comparison's reference group is highlighted in bold. The ASVs that are most representative of each variable in the model are shown; The p-value and the meaning of the interaction are shown in parentheses in the final column after all factors have been adjusted for.The patients' poor(mRS≥3) and good(mRS＜3) outcomes were identified during the 30-day and 90-day follow-up periods. *SAP* stroke-associated pneumonia; *mRS* modified Rankin Scale.

**Table S6 The specific taxa signature associated with clinical outcomes in VAP.**

| **Variable** | **Groups** | **ASV_S_ associated** | **P Value** | **Adjusted P Value** |
| --- | --- | --- | --- | --- |
| **30-day mortality** | **Yes/**No | *f__Campylobacteraceae* | 0.008 | 0.008(0.649) |
|  |  | *g__Campylobacter* | 0.008 | 0.008(0.649) |
|  |  | *p__Campilobacterota* | 0.008 | 0.008(0.642) |
|  |  | *c__Campylobacteria* | 0.008 | 0.008(0.642) |
|  |  | *o__Campylobacterales* | 0.008 | 0.008(0.642) |
|  |  | *f__Leptotrichiaceae* | 0.010 | 0.013(1.043) |
|  |  | *g__Leptotrichia* | 0.045 | 0.055(1.028) |
| **30-day mRS** | **Poor/**Good | *g__Peptostreptococcus* | 0.043 | 0.058(-1.290) |

The comparison's reference group is highlighted in bold. The ASVs that are most representative of each variable in the model are shown; The p-value and the meaning of the interaction are shown in parentheses in the final column after all factors have been adjusted for.The patients' poor(mRS≥3) and good(mRS＜3) outcomes were identified during the 30-day and 90-day follow-up periods. *VAP* ventilator-associated pneumonia; *mRS* modified Rankin Scale.

**Table S7 Univariate and multivariate logistic regression of the risk factors for a 90-day poor primary outcome of patients with SAP.**

| **Risk factor** | **Univariate analysis** | |  | **Multivariate analysis*** | |  | **Multivariate analysis^#^** | |
| --- | --- | --- | --- | --- | --- | --- | --- | --- |
|  | **OR(95% CI)** | **P value** |  | **Model 1 OR(95% CI)** | **P value** |  | **Model 2 OR(95% CI)** | **P value** |
| **P1** |  |  |  |  |  |  |  |  |
| ***p__Proteobacteria*** | 0.643(0.124-3.331) | 0.599 |  | NA | NA |  | NA | NA |
| ***p__Actinobacteriota*** | 11.819(1.175-118.917) | 0.036 |  | 9.486(1.296-69.444) | 0.027 |  | 18.529(1.609-213.447) | 0.019 |
| **P2** |  |  |  |  |  |  |  |  |
| ***p__Actinobacteriota*** | 3.338(0.685-16.265) | 0.136 |  | NA | NA |  | NA | NA |
| ***c__Actinobacteria*** | 2.451(0.603-9.964) | 0.210 |  | NA | NA |  | NA | NA |
| **Δ** |  |  |  |  |  |  |  |  |
| ***p__Actinobacteriota*** | 0.409(0.071-2.345) | 0.316 |  | NA | NA |  | NA | NA |
| ***c__Actinobacteria*** | 0.477(0.097-2.341) | 0.362 |  | NA | NA |  | NA | NA |

* Model 1 adjusted by SOFA, APACHE II, initial GCS score and length of ICU stay.

# Model 2 adjusted by age, initial NIHSS score, enteral nutrition, atrial fibrillation, dysphagia, and NLR(neutrophil to lymphocyte ratio).

P1= log-transformed average(before diagnosis of SAP samples); P2= log-transformed average(after diagnosis of SAP samples); Δ=P2-P1.

**Table S8 Logistic regression analysis of risk factors associated with 30-day mortality in SAP group.**

| **Risk factor** | **Univariate analysis** | |
| --- | --- | --- |
|  | **OR（95% CI）** | **P value** |
| **P1** |  |  |
| ***p__Proteobacteria*** | 1.042（0.165-6.580） | 0.965 |
| **P2** |  |  |
| ***p__Proteobacteria*** | 1.099（0.133-9.072） | 0.930 |
| **Δ** |  |  |
| ***p__Proteobacteria*** | 0.776（0.122-4.948） | 0.789 |

P1= log-transformed average(before diagnosis of SAP samples); P2= log-transformed average(after diagnosis of SAP samples); Δ=P2-P1.

**Table S9 Logistic regression analysis of risk factors associated with 30-day mortality in VAP group.**

| **Risk factor** | **Univariate analysis** | |
| --- | --- | --- |
|  | **OR（95% CI）** | **P value** |
| P1 |  |  |
| *p__Campilobacterota* | 19.011（0.241-1497.971） | 0.186 |
| *c__Campylobacteria* | 19.011（0.241-1497.971） | 0.186 |
| *o__Campylobacterales* | 19.011（0.241-1497.971） | 0.186 |
| *f__Campylobacteraceae* | 19.602（0.243-1578.715） | 0.184 |
| *f__Leptotrichiaceae* | 277.186（0.164-4.69E+05） | 0.138 |
| *g__Campylobacter* | 19.602（0.243-1578.715） | 0.184 |
| *g__Leptotrichia* | 7.582（0.294-195.282） | 0.222 |
| P2 |  |  |
| *p__Campilobacterota* | 26.374（0.087-7996.061） | 0.262 |
| *c__Campylobacteria* | 26.374（0.087-7996.061） | 0.262 |
| *o__Campylobacterales* | 26.374（0.087-7996.061） | 0.262 |
| *f__Campylobacteraceae* | 25.086（0.077-8124.329） | 0.275 |
| *f__Leptotrichiaceae* | 8.41E+07（8.55E-7-8.27E+21） | 0.267 |
| *g__Campylobacter* | 25.086（0.077-8124.329） | 0.275 |
| *g__Leptotrichia* | 526.874（0.030-9.28E+06） | 0.209 |
| Δ |  |  |
| *p__Campilobacterota* | 0.316（0.022-4.457） | 0.393 |
| *c__Campylobacteria* | 0.316（0.022-4.457） | 0.393 |
| *o__Campylobacterales* | 0.316（0.022-4.457） | 0.393 |
| *f__Campylobacteraceae* | 0.299（0.021-4.333） | 0.376 |
| *f__Leptotrichiaceae* | 0.932（0.139-6.264） | 0.943 |
| *g__Campylobacter* | 0.299（0.021-4.333） | 0.376 |
| *g__Leptotrichia* | 1.239（0.193-7.957） | 0.821 |

P1= log-transformed average(before diagnosis of VAP samples); P2= log-transformed average(after diagnosis of VAP samples); Δ=P2-P1.

**Supplementary Figures**

**
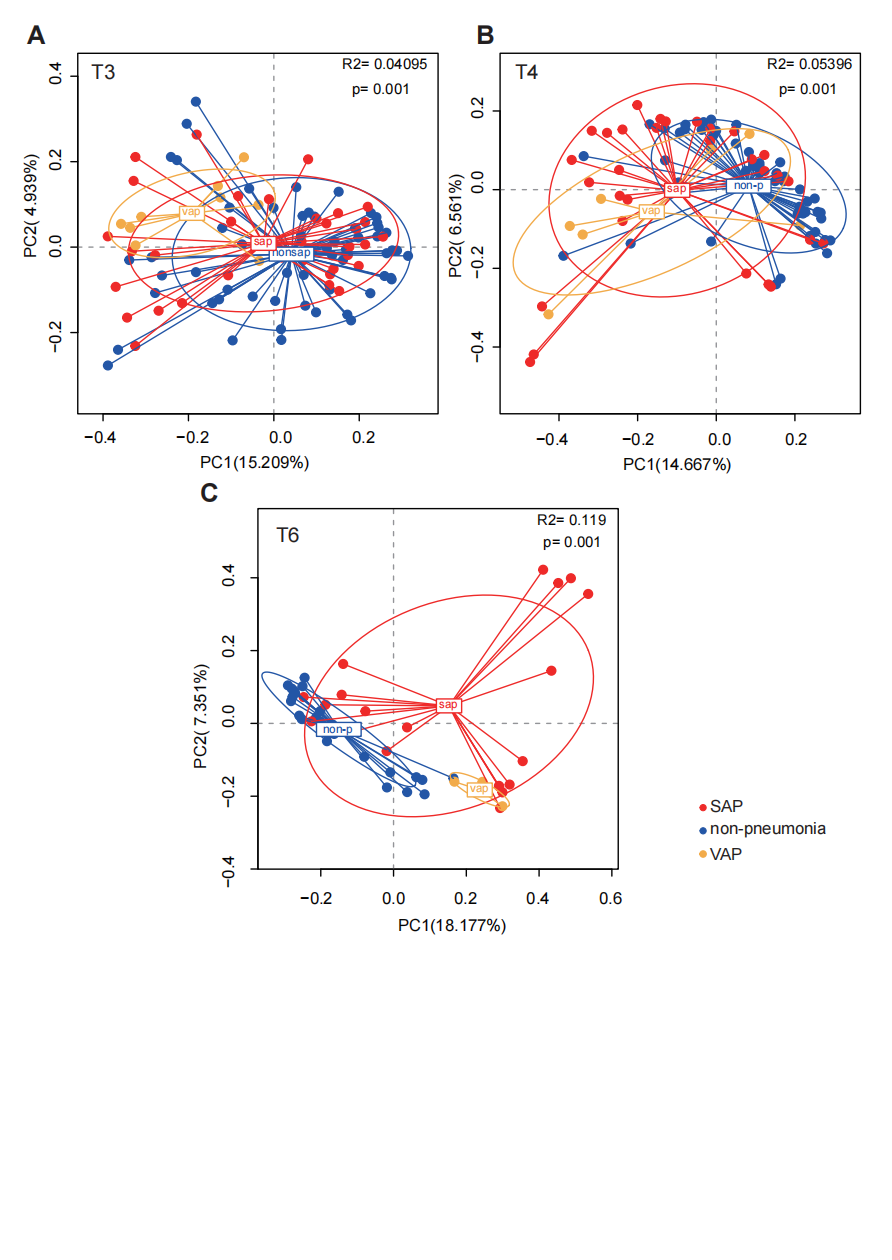
**

**Figure S1**. PCoA plots based on bray-curtis distances is drawn to display the dynamic differences among three groups at T3,T4 and T6. Each point represents the composition of the intestinal microbiota of one participant. *SAP* stroke-associated pneumonia; *VAP* ventilator-associated pneumonia.


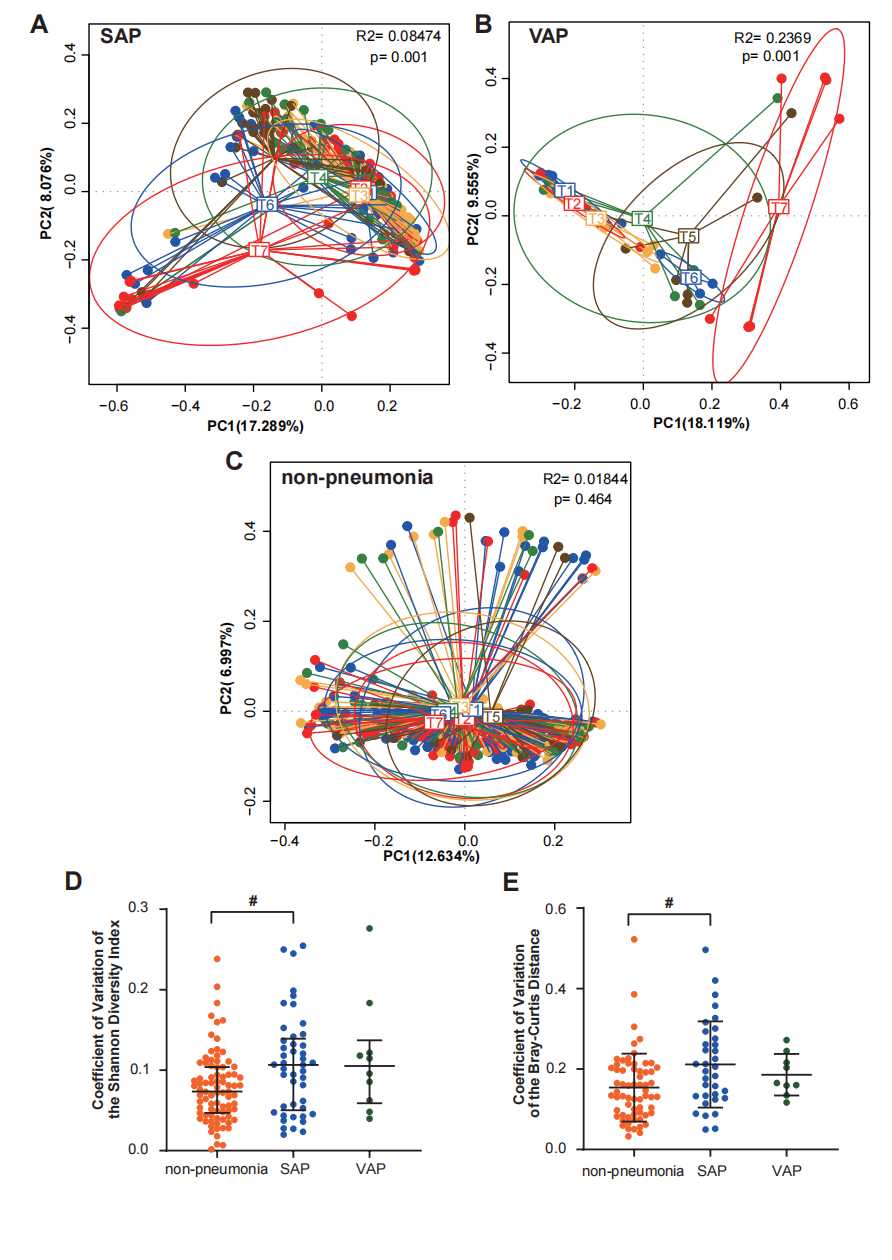


**Figure S2**. **A,B,C** PCoA plot based on bray-curtis distances illustrating the alter patterns for each groups respectively. **D,E** The CVs of the shannon diversity and bray-curtis distances. P values comparing the different groups were calculated using Wilcoxon tests, p <0 .05, *among three group, # SAP compared with non. *SAP* stroke-associated pneumonia; *VAP* ventilator-associated pneumonia; *CV* the coefficient of variation.


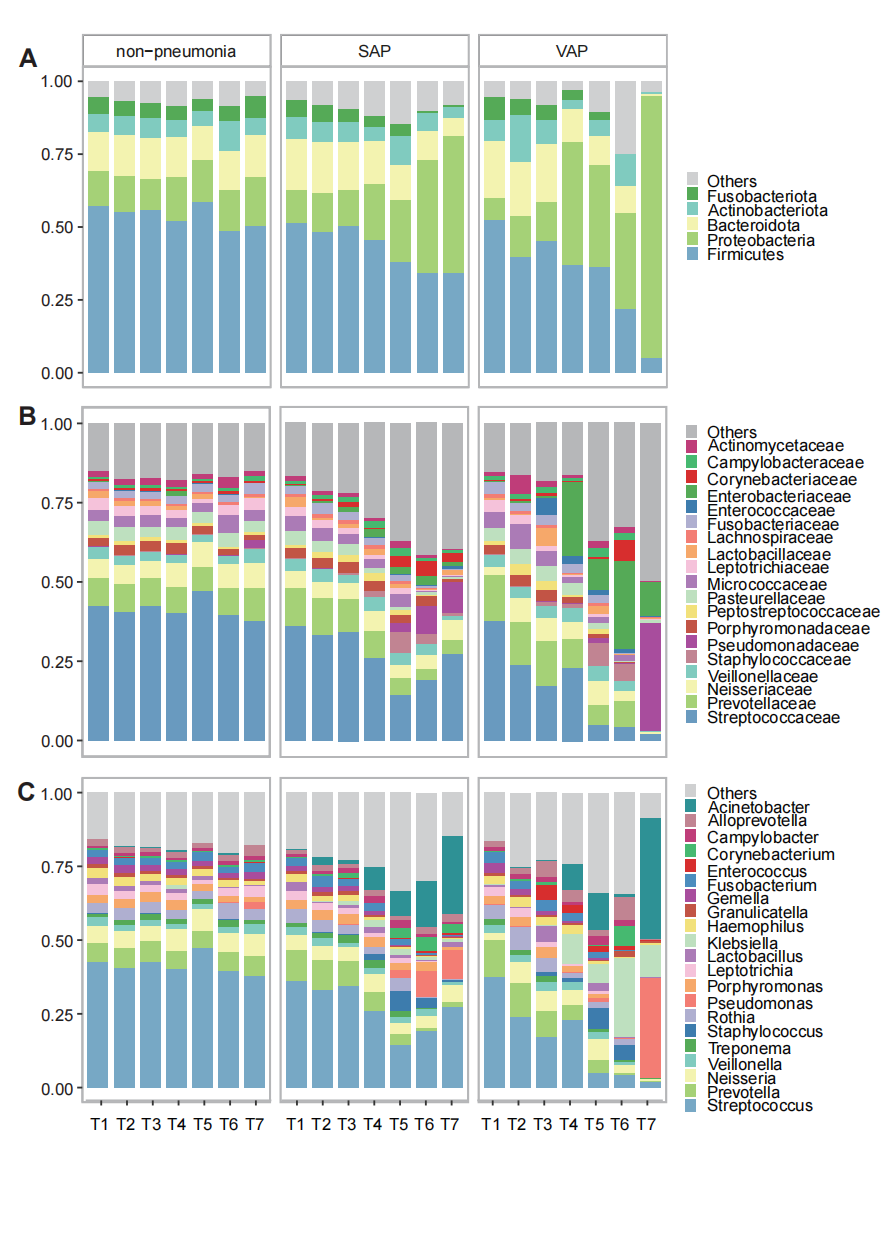


**Figure S3** Comparative taxonomic profiles showed significantly dynamic differences in abundance among three groups. The overall composition of the oral microbiota at the phylum(**A**), family(**B**) and genus(**C**) levels was determined for each groups and each time points.


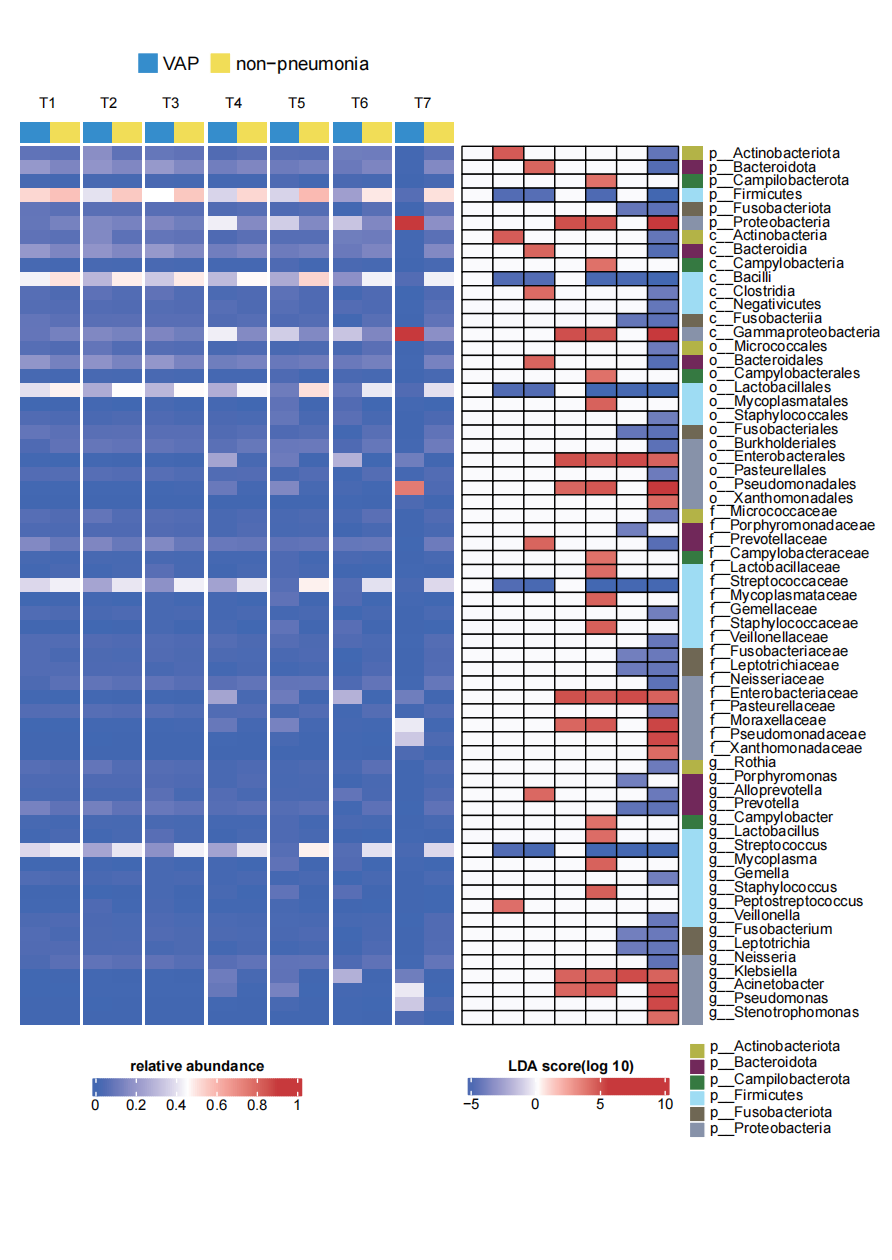


**Figure S4.** The LEfSe analysis shows that the representation of the various bacterial taxa changed over the time points between VAP and non-pneumonia. Only taxa with a statistically significant LDA score (log10) >4 are shown. The heat map on the left shows the relative abundance of the taxa, and the heat map on the right shows the LDA scores. *LEfSe* Linear discriminant analysis effect size.


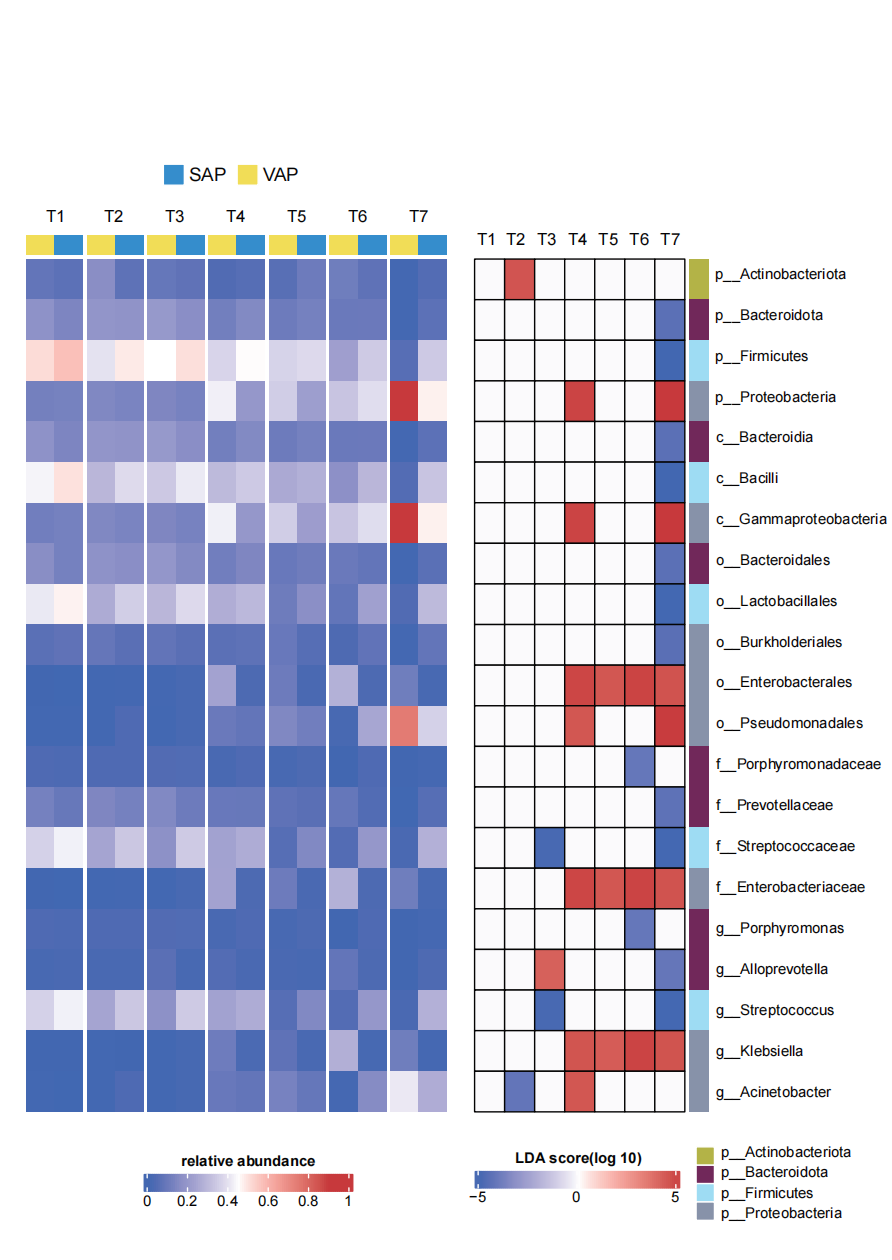


**Figure S5.** The LEfSe analysis shows that the representation of the various bacterial taxa changed over the time points between VAP and SAP. Only taxa with a statistically significant LDA score (log10) >4 are shown. The heat map on the left shows the relative abundance of the taxa, and the heat map on the right shows the LDA scores. *LEfSe* Linear discriminant analysis effect size.


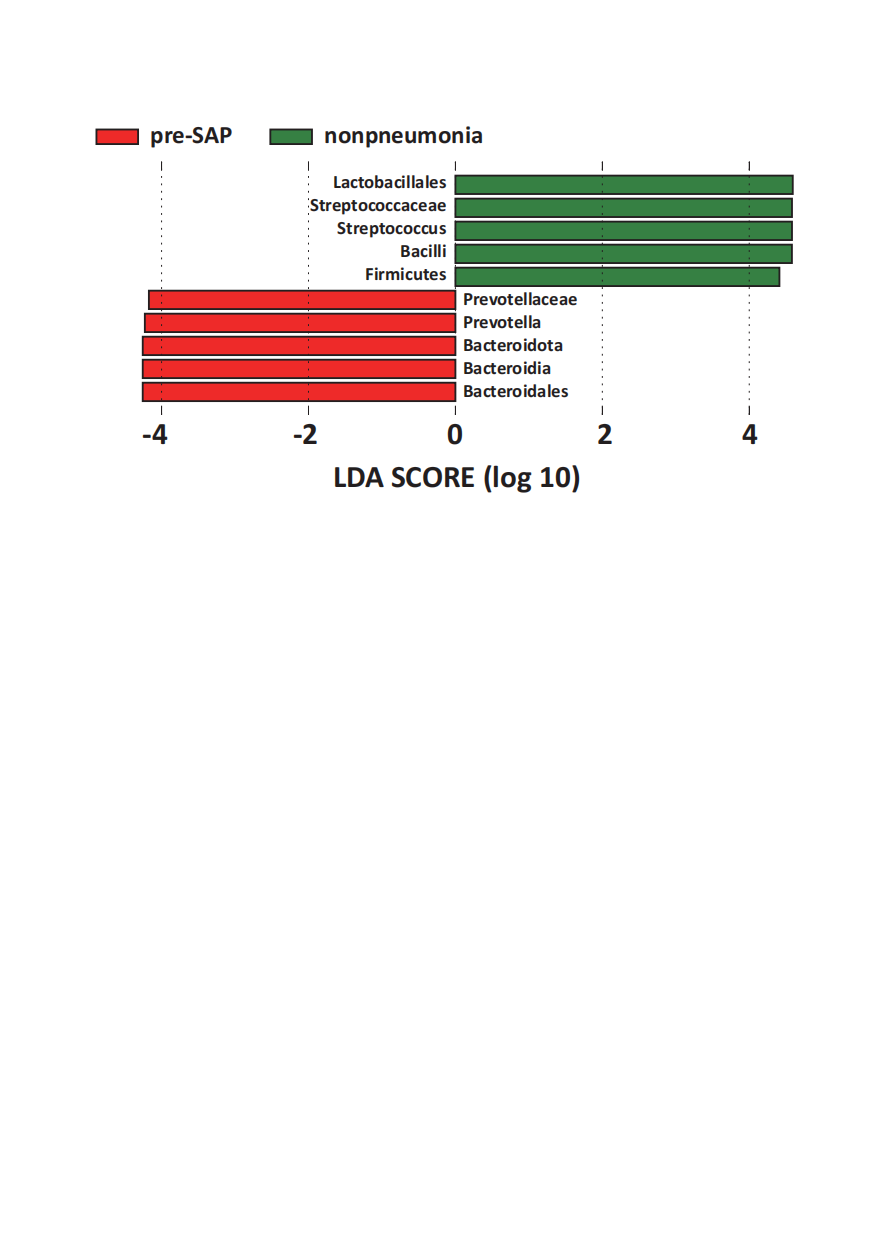


Figure S6. The LEfSe analysis shows that the representation of the various bacterial taxa changed between nonpneumonia and pre-SAP.


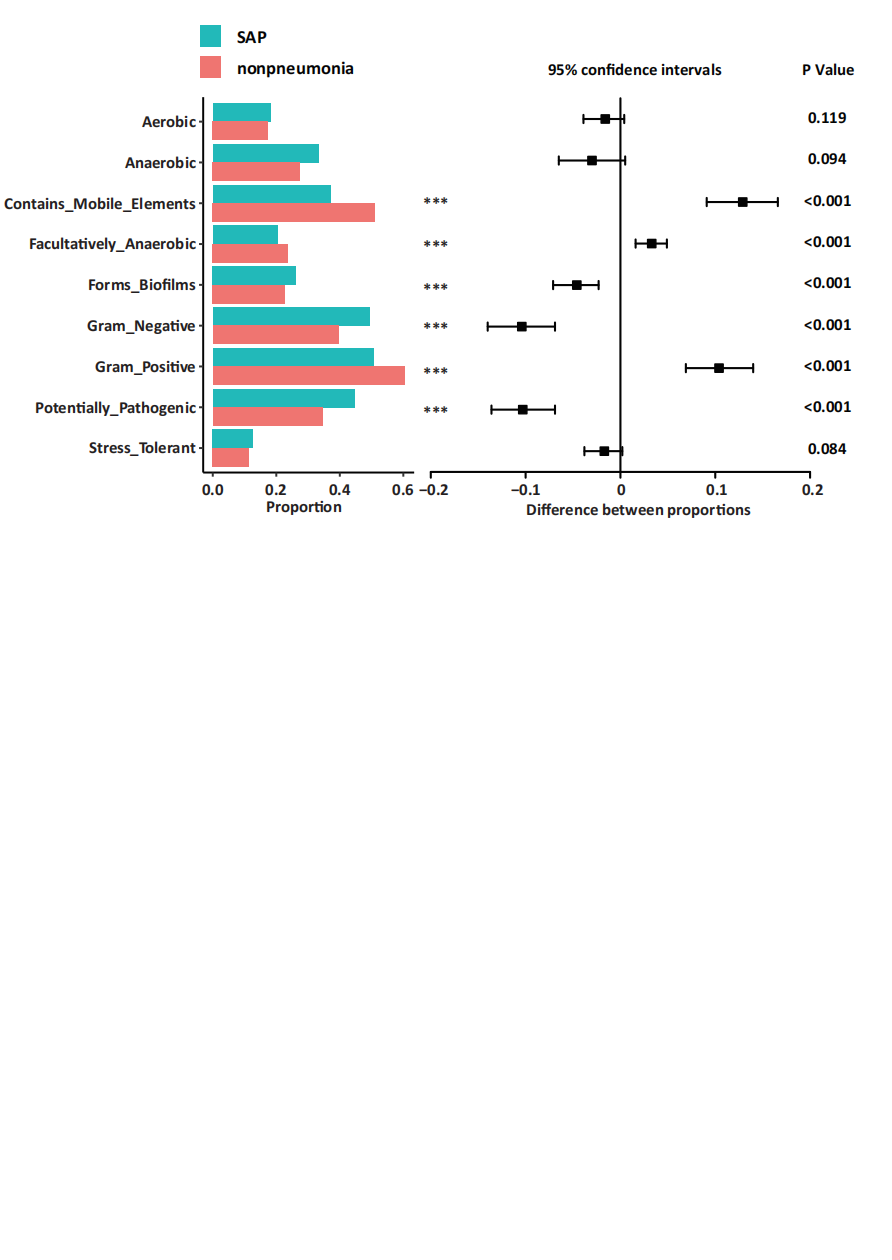


Figure S7. Microbial phenotype prediction by BugBase. The different microbial compositions between SAP and nonpneumonia.


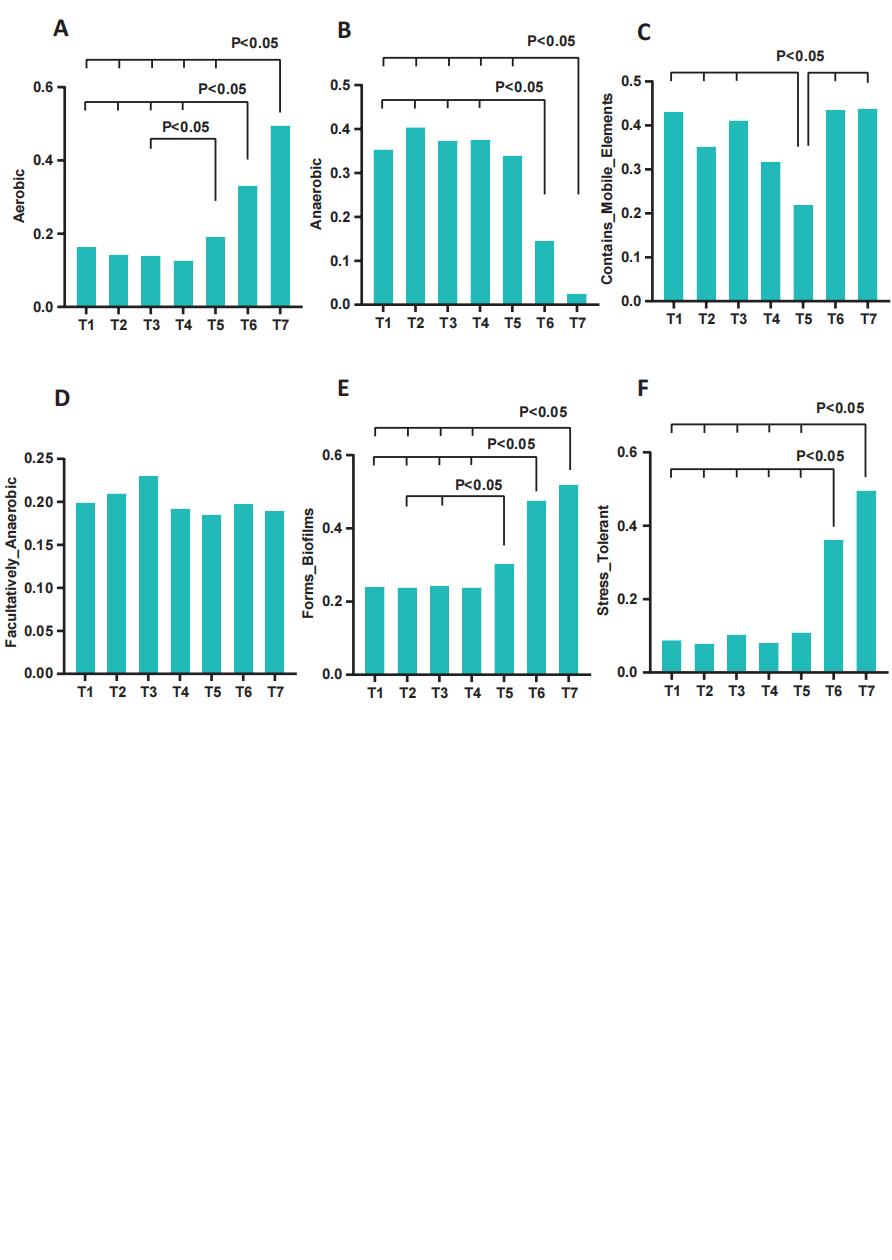


**Figure S8.** Microbial function prediction by BugBase. (A,B,C,D,E,F) Dynamic characteristics of oral microbiota in SAP. The connection of fold lines represents a significant difference between different time points.


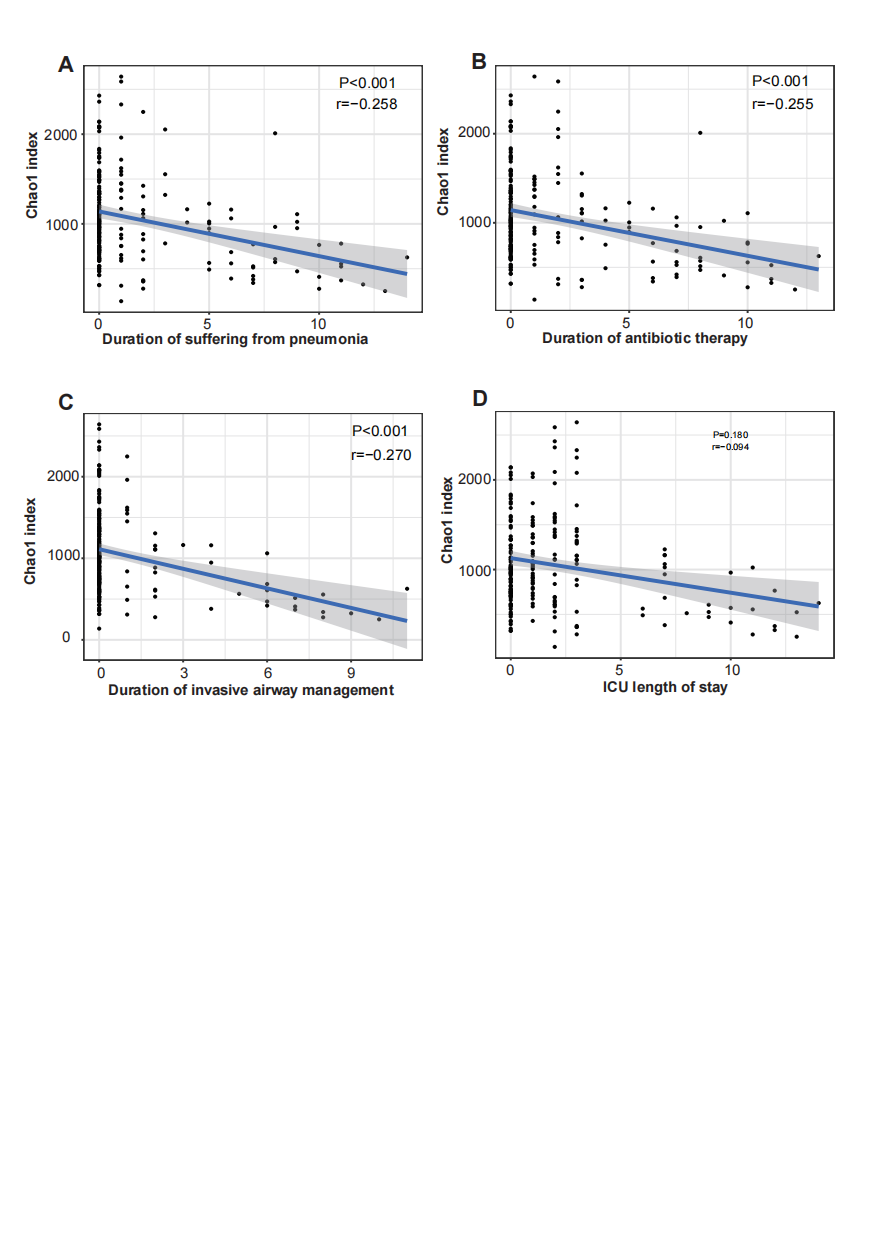


**Figure S9.** The invasive and pharmacological medical care treatments influence oral microbiota after stroke. The duration of suffering from pneumonia(**A**), duration of antibiotic therapy(**B**), duration of invasive airway management(**C**) and ICU length of stay(**D**) were negatively associated with Chao1 Index.


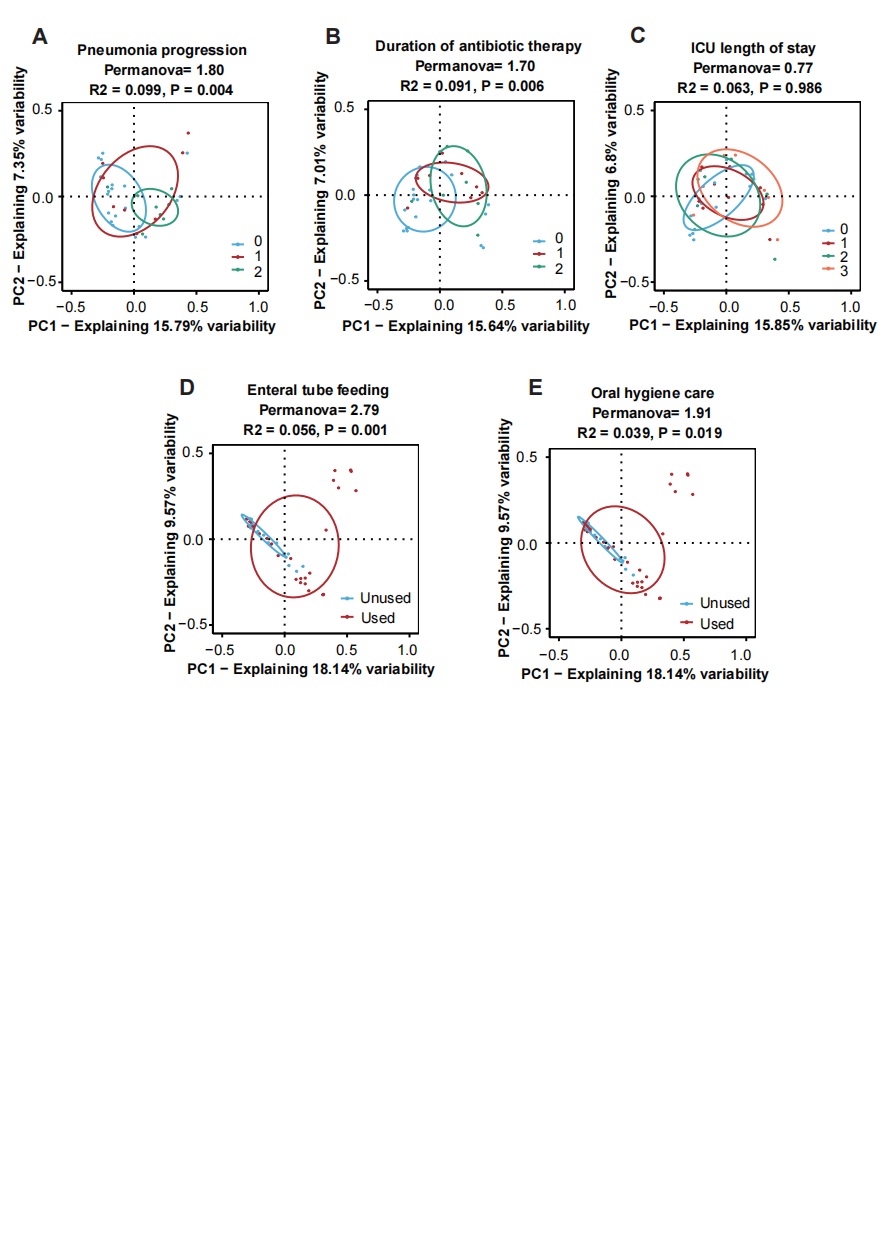


**Figure S10.** PCoA of multidimensional data is drawn to display changes in microbial communities according to major variables in VAP: pneumonia progression(**A**), duration of antibiotic therapy(**B**), ICU length of stay(**C**), Enteral tube feeding(**D**) and Oral hygiene care(**E**). The x- and y-axes represent the two most informative PCs of the PCoA, and marginal boxplots describe the distribution of those values for the different groups. Color legends represent the respective variables under analysis. The number refer to the number of days in **A,B,C**. The results of the PERMANOVA to compare dissimilarity indexes among samples are shown on top of plots accordingly. *PCs* principal coordinates; *PERMANOVA* permutation-based test.


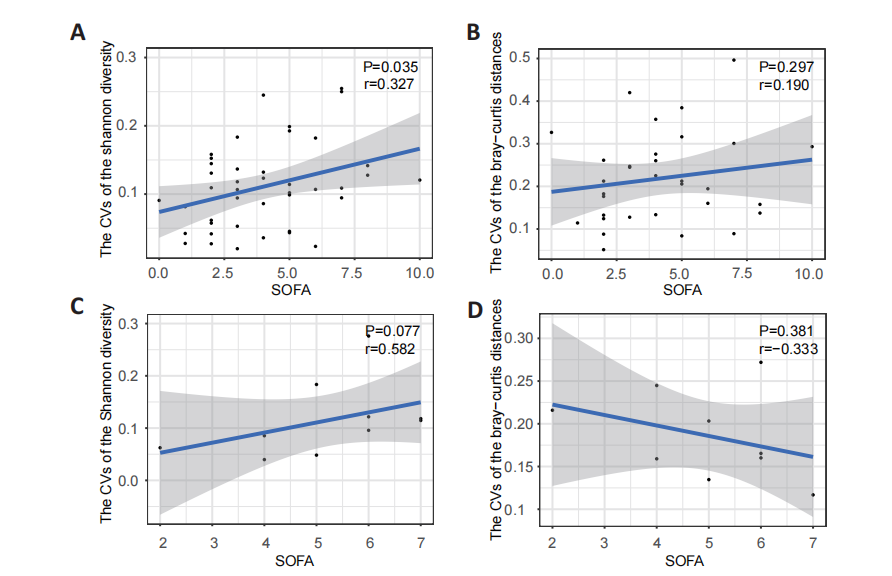


**Figure S11.** The CVs of the shannon diversity and bray-curtis distances are associated with SOFA in SAP (**A,B**)and VAP groups(**C,D**)respectively.


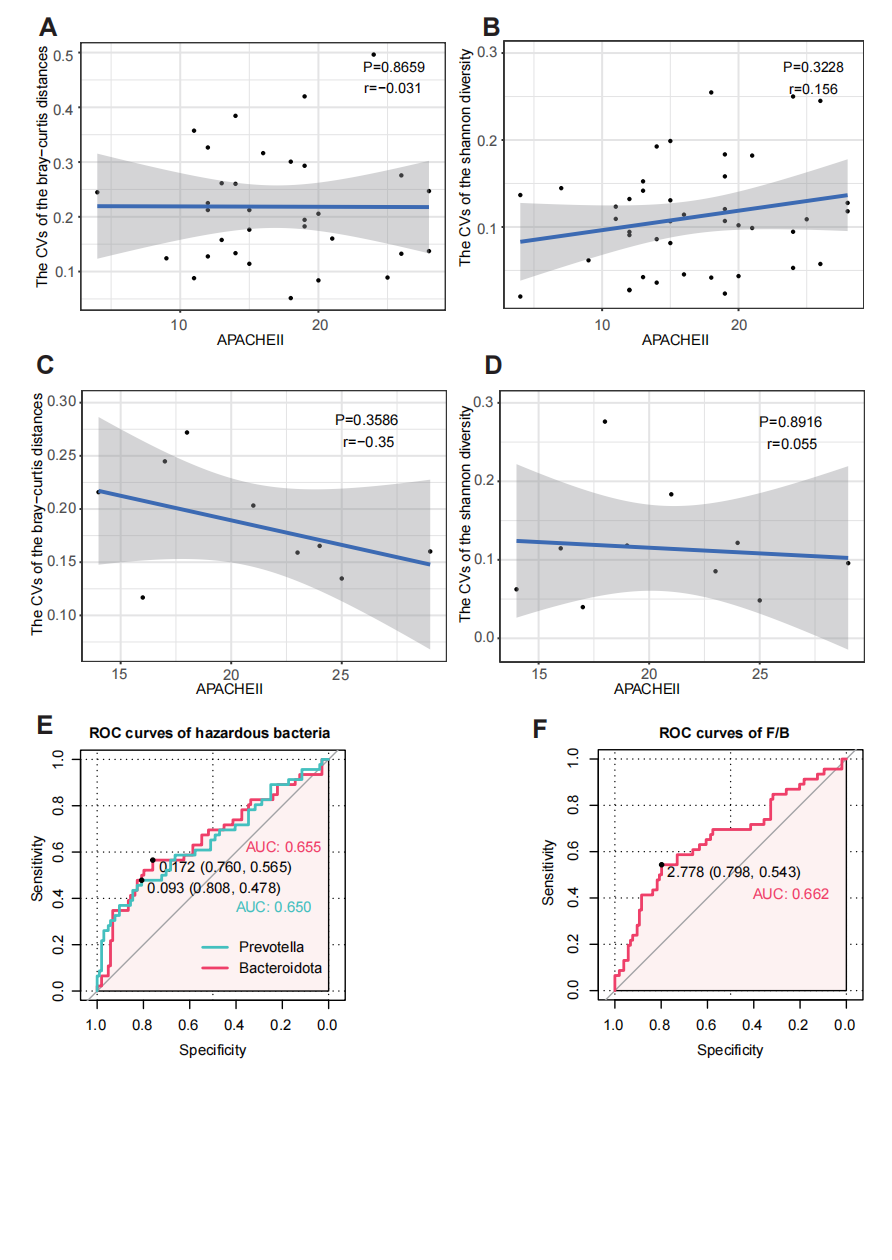


**Figure S12.** The CVs of the shannon diversity and bray-curtis distances are associated with APACHEⅡ in SAP (**A,B**)and VAP groups(**C,D**)respectively.


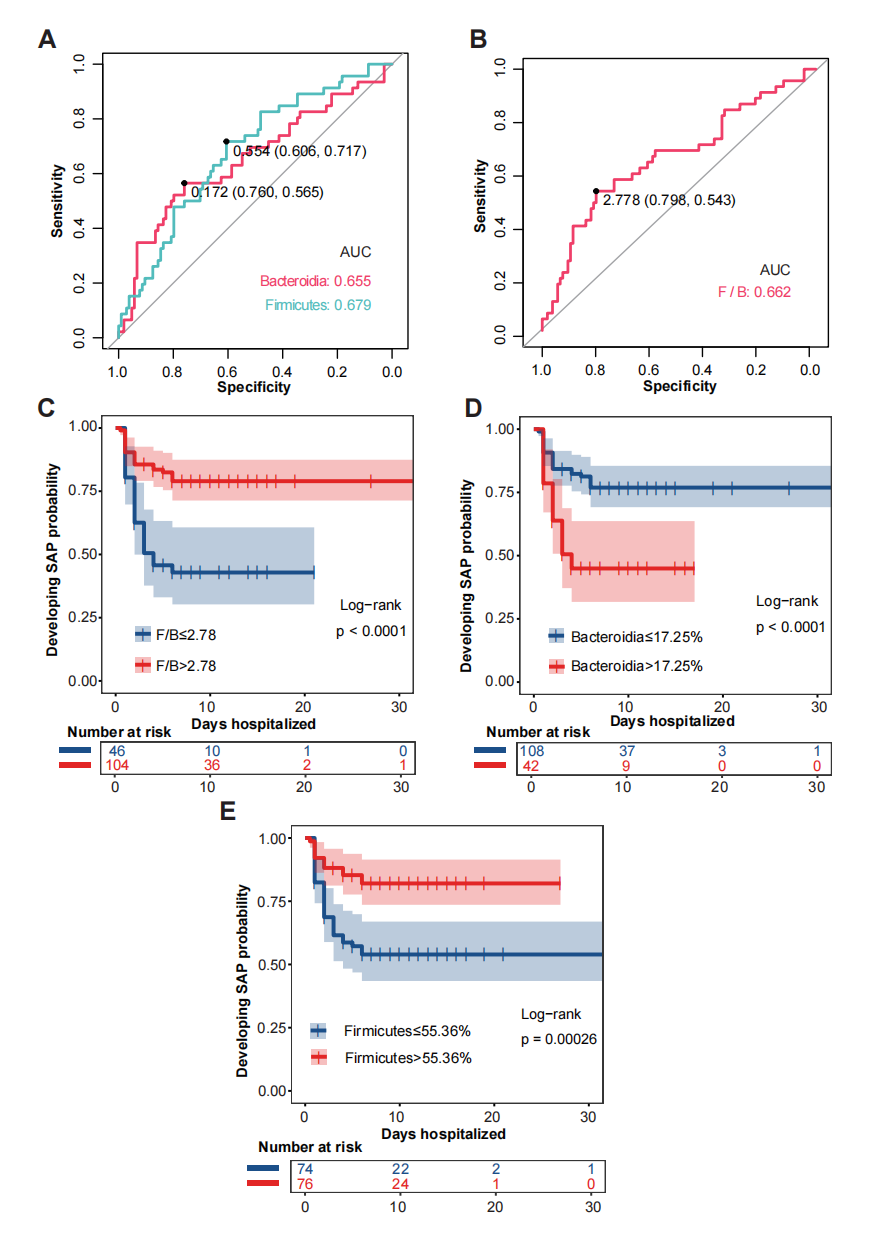


**Figure S13.** The relative abundance of specific taxa associated with SAP. ROC curve analyses for derivation of thresholds of *Bacteroidota ,**Firmicutes* relative abundance(**A**) and F/B ratio(**B**). Kaplan-Meier curves for developing SAP probability during hospitalization stratified by thresholds of *Bacteroidota ,* *Firmicutes* relative abundance (**D, E**) and F/B ratio(**C**). *P* values were derived from the log-rank test. *ROC* Receiver operator characteristic.


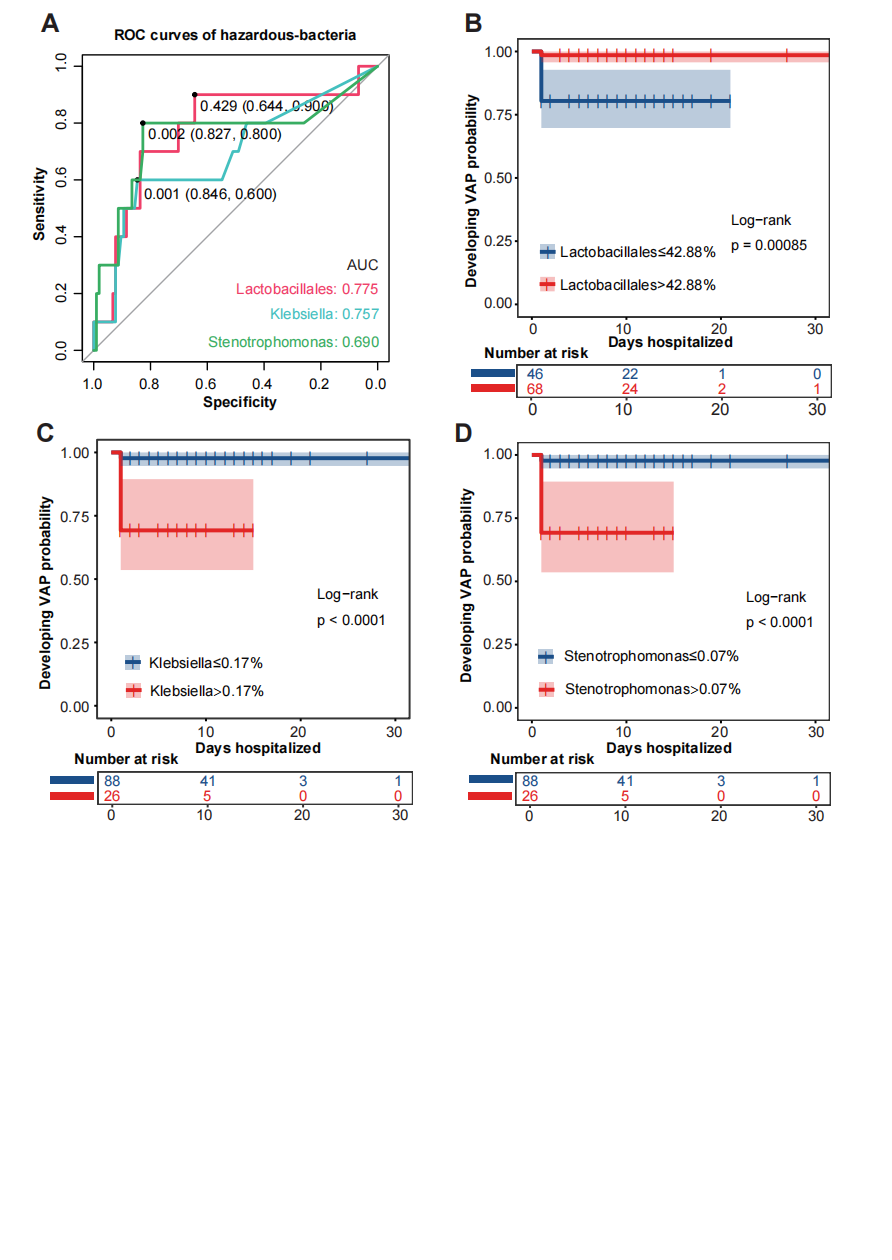


**Figure S14.** The relative abundance of specific taxa associated with VAP. ROC curve analyses for derivation of thresholds of protective-bacteria (*Lactobacillales*) relative abundance(**A**) and hazardous-bacteria (*Klebsiella , Stenotrophomonas*) (**A**). Kaplan-Meier curves for developing VAP probability during hospitalization stratified by thresholds of protective-bacteria (*Lactobacillales*) relative abundance(**B**) and hazardous-bacteria (*Klebsiella , Stenotrophomonas*) (**C,D**). *P* values were derived from the log-rank test. *ROC* Receiver operator characteristic.
